# The Elephant Trunk Skin Inspires a Highly Sensitive and Deformable, Yet Robust, Armor Skin

**DOI:** 10.1002/advs.74963

**Published:** 2026-03-25

**Authors:** Jun Chang Yang, Petr Trunin, Behnam Kamare, Lucia Beccai

**Affiliations:** ^1^ Soft BioRobotics Perception Lab Istituto Italiano di Tecnologia Genova Italy; ^2^ The Open University Affiliated Research Centre at Istituto Italiano Di Tecnologia (ARC@IIT) Istituto Italiano di Tecnologia Genova Italy; ^3^ The Biorobotics Institute Scuola Superiore Sant'Anna Pontedera Italy

**Keywords:** armor skin, bio‐inspired skin, elephant trunk skin, fold structure, puncture resistance, tactile sensing

## Abstract

Soft artificial skins attract significant attention due to the inherent high deformability and tactile sensing functions, making them highly beneficial for skin‐attachable electronics and robotics. However, they have limited mechanical robustness, which is a requirement for real‐world applications. Therefore, an evolution to soft “armor skins” is necessary, where deformation, tactile sensitivity, and ruggedness co‐exist. We look at the elephant trunk skin as a remarkable model that due to its unique hierarchical structure deforms freely accommodating the trunk versatile motions while maintaining mechanical robustness, providing protection while also allowing for highly sensitive touch. Inspired by this we present the elephant trunk‐inspired armor skin with tactile sensing (ETATS), which exhibits high deformability, resistance to puncture and tear, tactile and strain sensing capability. The ETATS consists of a folded soft substrate reinforced with aligned fibers, capable of 60% stretchability of 40% compressibility in lateral direction, combined with a rigid hexagonal island array for strong puncture resistance (97.5 N). Morphology‐based decoupling of strain and pressure enables large‐area pressure and lateral strain sensing in real‐time by embedded optical waveguides. Our strategy maximizes the stretchability, protective capability, and sensing performance of electronic skin, making it highly beneficial for applications in both humans and robots.

## Graphical abstract

The study presents the elephant trunk‐inspired armor skin with tactile sensing (ETATS). The ETATS architecture stretches and compresses while resisting puncture and tear, and it enables optical detection of pressure and lateral strain. Morphology‐based decoupling of strain and pressure paves the way to sensitive and deformable, yet robust, soft‐robotic skins to deploy in the real environment.

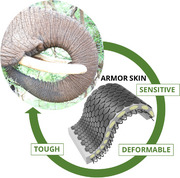

## 1 Introduction

Soft robots are becoming increasingly sophisticated and dexterous, and they are beginning to transition from controlled laboratory settings to unstructured real‐world environments [1, 2, 3, 4]. In these contexts, they not only require sensing capabilities (e.g., tactile sensing) to perceive their surroundings, but they also must be rugged to repel mechanical damage. As a result, new performance requirements have emerged: soft robots must be not only flexible, adaptable, and sensor‐integrated, but also mechanically robust and well protected. Conventional protection strategies, such as rigid external shells, are incompatible with the inherent compliance and deformability of soft robotic systems. To address this, several approaches have been proposed, including the enhancement of mechanical properties through functional materials [5, 6], the incorporation of rigid layers [7, 8], and the development of self‐healing materials [9, 10]. Among these, self‐healing systems enable autonomous recovery from mechanical damage such as punctures, cuts, and tears; however, they typically offer limited immediate protection and require time to restore functionality.

Continuum arms and manipulators are of main interest in soft robotics, and for their development and deployment in the environment researchers have long been inspired by the boneless elephant trunk [11, 12, 13], which muscular anatomy and prehensile capabilities have also recently been studied [14, 15]. However, the interaction with the environment is mediated by the trunk's skin [16, 17] that, noteworthy, represents a truly natural sensitive armor. It provides protection but at the same time it allows the multi‐degree‐of‐freedom movements of the trunk (i.e., extension, compression, twisting, bending), and is highly sensitive to touch [18, 19] enabling exploration and manipulation tasks. This distinctive combination of strength, flexibility, and sensitivity makes the elephant trunk skin an intriguing model for advanced “armor skin” designs.

Biologically inspired armor skins have been previously designed after natural structures such as fish scales [20, 21, 22, 23], armadillo shells [7, 20], crocodile armor [24], and snake scales [25], mainly combining a soft, deformable substrate with rigid surface elements (e.g., scales or domes). These hybrid architectures provide significant resistance to punctures and lacerations, even under deformation. Nonetheless, existing designs predominantly address bending deformation and remain limited in their ability to accommodate stretching and compression. Furthermore, tactile sensing is mostly missing, except for case [25] in which the skin provides pressure feedback but offers only partial protection when stretched.

Indeed, designing an armor skin that is sensitive to tactile stimuli is an open challenge, involving contrasting requirements because it must be compliant enough to deform and promote tactile sensitivity, but at the same time it must be rugged and endure the mechanical interaction with the outside world.

**FIGURE 1:**
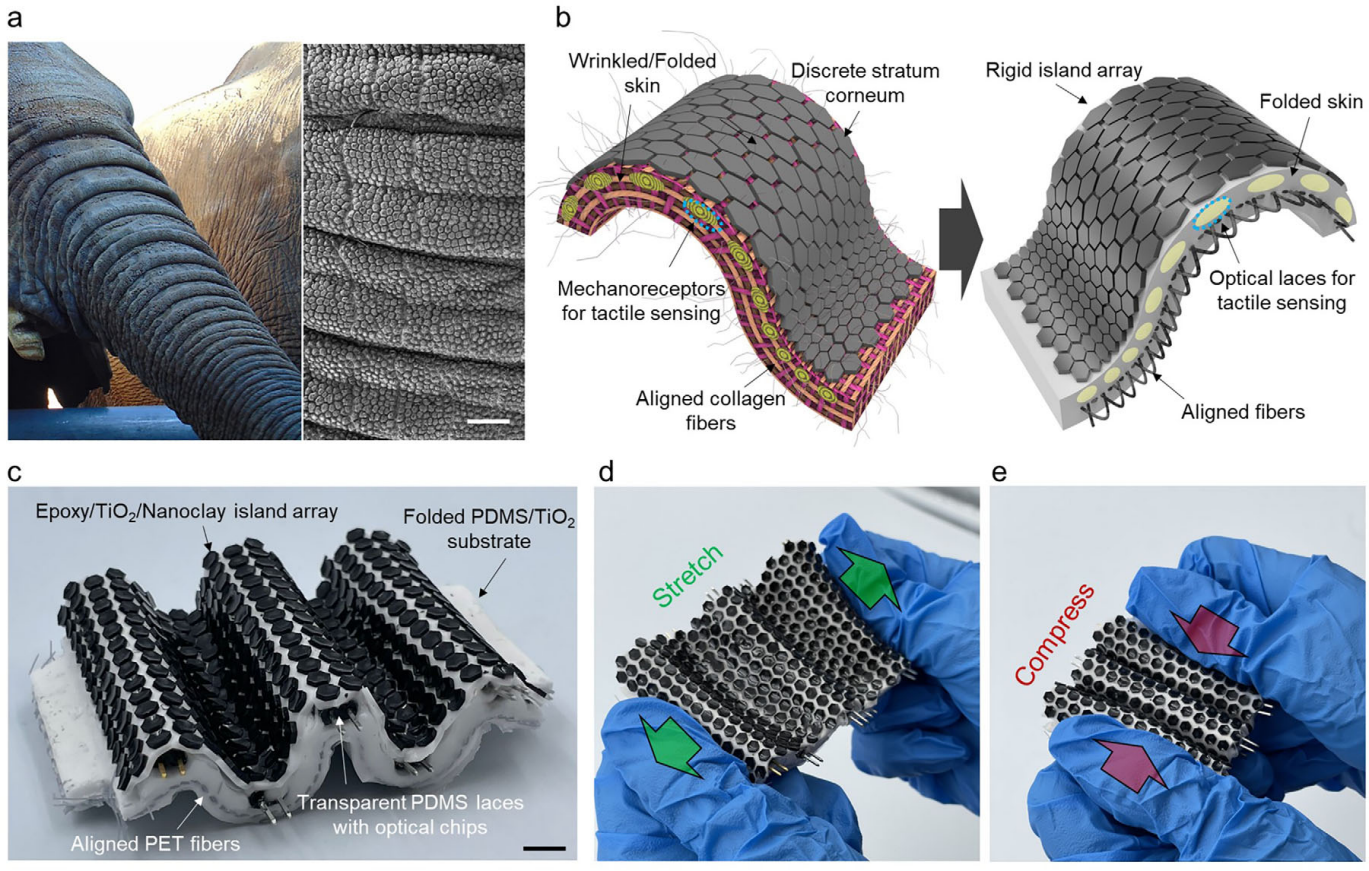
Design of elephant trunk‐inspired armor skin with tactile sensing (ETATS). a) Photograph of an African elephant trunk and close‐up of the skin superficial structure with folds, wrinkles and crevices (scale bar is 5 mm). The animal was observed at the ZooSafari, Fasano, Italy. b) Simplified schematic of the natural skin (left), highlighting the island‐shaped stratum corneum, wrinkled/folded structure, aligned collagen fibers, and mechanoreceptors for tactile sensing, compared to the ETATS design (right), which mimics these features with a superficial rigid island‐like array, optical waveguide‐based tactile sensors, and aligned PET fibers. c–e) Photographs of the ETATS when is (c) relaxed, (d) stretched, and (e) compressed. Scale bar in (c) is 6 mm.

We address this challenge by taking inspiration from the elephant trunk skin (Figure 1a. Although a complete anatomical description of the elephant skin is still lacking, based on the results found in the literature (including our previous study [16]), and for the sole purpose of this work, we can consider a simplified schematic of the skin cross‐section in Figure 1b (left), comprising: 1) a folded and wrinkled structure that allows the thick skin (≈15 mm) to stretch (tensile strain of 30%) and compress (compressive strain of 30%) [17]; 2) the outermost layer of skin, the stratum corneum (part of the epidermis), characterized by a discrete island‐like structure [16] and composed of highly keratinized material that forms a mosaic pattern (Figures S1a,b and S2); 3) nerve endings (or mechanoreceptors) providing tactile sensitivity [26, 27, 28]; and, 4) collagen fibers (Figure S1c) [29] entangled in the dermis and putatively contributing to support the skin [30].

The proposed elephant trunk‐inspired armor skin with tactile sensing (ETATS), Figure 1b (right), comprises the following main components: 1) a folded substrate enabling the skin (60 mm × 50 mm × 5.8 mm) to stretch uniaxially (tensile strain of up to 60%) and compress (compressive strain of up to 40%); 2) an hexagonal‐shaped island array showing high resistance to puncture and cutting (critical force of 97.5 N); 3) optical waveguide‐based sensor array that can detect pressure (<250 kPa) both during skin stretching and compression, and lateral strain (‐30%–+30%)—where we make use of the morphology (i.e., folding) to discriminate pressure from strain; and, 4) the skin with aligned fibers exhibiting 4.3 times higher toughness than the fiber‐free skin, showing resistance to tear.

Overall, compared to the literature (Table S1), the ETATS concept provides new capabilities for armor‐like skin in terms of mechanical performance (i.e., toughness and deformability), while introducing the sensing capability, quite overlooked so far in armor skins. As shown in Figure 1c, the optical tactile sensing is implemented by transparent waveguides and optical chips (photoemitters and photoreceivers) embedded in a folded polymer substrate with reflective shielding. Polydimethylsiloxane (PDMS) and a PDMS/TiO_2_ composite are employed to fabricate the transparent waveguides and polymer substrate, respectively. The aligned polyethylene terephthalate (PET) fibers are located 1 mm below the folded PDMS/TiO_2_ skin, while epoxy‐based separate domes (named rigid island array) cover the top of the skin. The fabrication process of ETATS is optimized to ensure high yield and reliability, given the various integrated components (see Figure S3 and Materials and Method Section). Figure 1d,e and Figure S4 depict the high deformability of ETATS, together with its rugged nature providing protection.

## 2 Results

### 2.1 Enabling Mechanical Robustness in the Soft Artificial Skin

**FIGURE 2:**
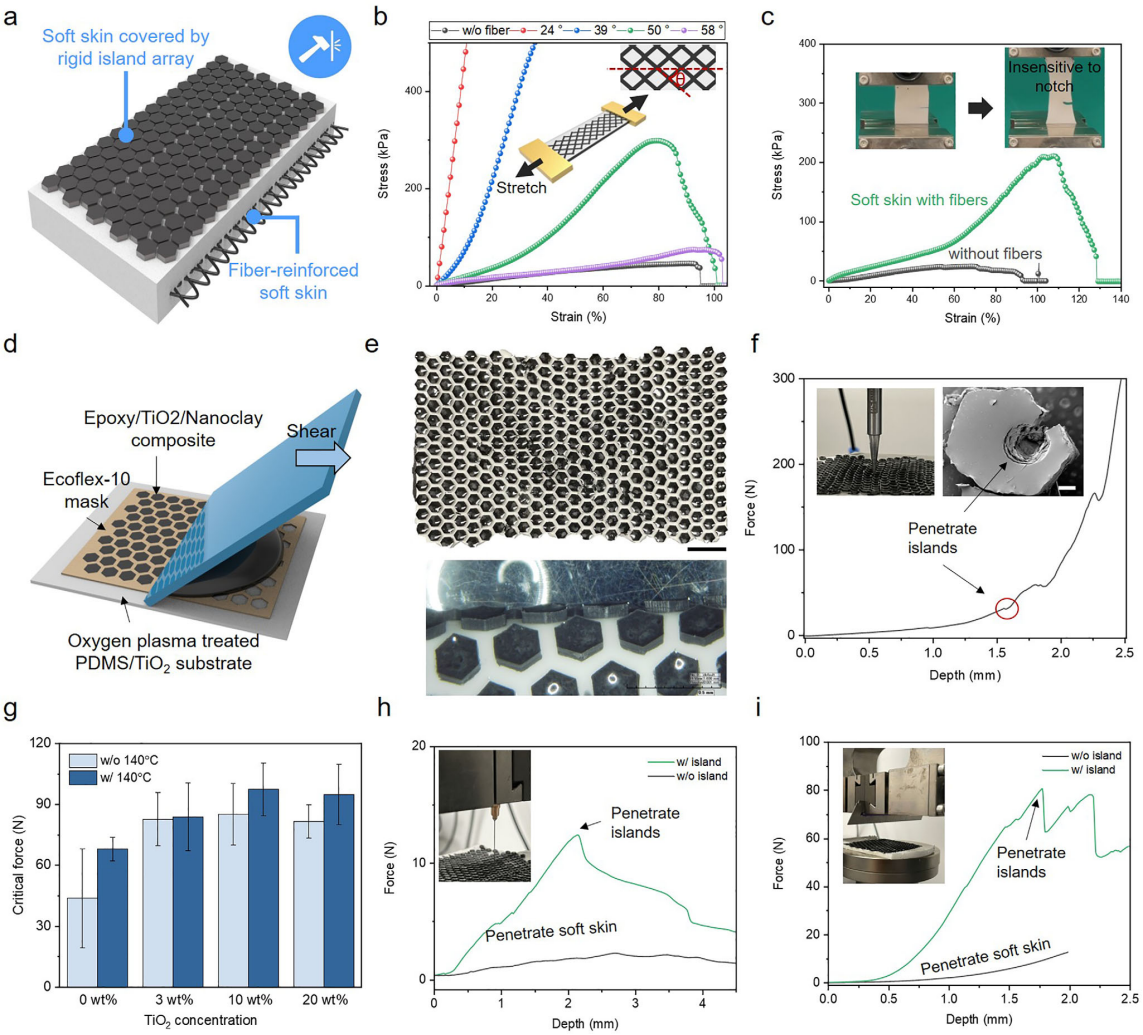
Fiber‐reinforced soft skin and rigid island array for mechanical robustness of ETATS. a) Schematic illustration of fiber‐reinforced soft skin and rigid island array for mechanical robustness. b) Stress versus strain for PDMS/TiO_2_ composite‐based skin with fibers aligned at different angles. c) Stress versus strain for notched soft skin with and without fibers aligned at 50°. The insets show aligned fibers at 50° effectively suppress crack propagation from the notch. d) Fabrication process of rigid island array via mask‐assisted blade coating: (i) epoxy/TiO_2_/nanoclay composite solution is blade‐coated on the oxygen plasma‐treated soft skin covered with a mask; (ii) the solution is cured by heating to form hydrogen bonding between the soft skin and epoxy islands. e) Top: photographs of large‐area rigid island array on soft skin; bottom: optical microscope image of the island array without defects. Scale bar in the top image is 1 cm. f) Force versus depth for epoxy island without TiO_2_ during puncture test. The insets show that islands are being penetrated. Scale bar in the left inset is 500 µm. g) Puncture resistance for islands with the different TiO_2_ concentration and post‐curing. h) Force versus depth for soft skin with and without islands during needle puncture test. i) Force versus depth for soft skin with and without islands under a cutting test.

We investigated two main aspects to enable mechanical robustness in the ETATS, (Figure 2a): (1) the soft skin fiber reinforcement, to prevent tearing caused by the propagation of puncture‐induced cracks during excessive or repetitive deformation (mainly stretching); (2) a reliable covering of the exposed top of the skin with a rigid island array (inspired by the natural elephant skin [31]) to provide overall protection against damaging from sharp objects. Extensive research has been conducted to enhance mechanical robustness of soft materials particularly focusing individually on their toughness [32, 33] or puncture and cut resistance [7, 24]. However, there is still a need of effective solutions to enhance toughness and resistance to puncture and cut, while not impairing the inherent deformation capability of soft materials.

In this study, we present an optimized architecture for a PDMS/TiO_2_ composite (hereafter named as “soft skin” Figure 2a)—a cladding material that is soft and stretchable, in which TiO_2_ particles are incorporated to form a reflective coating [34, 35], as discussed in detail in Section 2.3. Specifically, when such composite is used to build a soft substrate, it is vulnerable to tearing and to puncture because of its low toughness.

The incorporation of fibers into polymer matrices (e.g., elastomer, hydrogel, epoxy) has previously been reported to enhance their mechanical properties (e.g., modulus, toughness, crack resistance) [36, 37, 38, 39]. Herein, reticular‐aligned PET fibers are integrated in the soft skin (70 mm × 30 mm × 2 mm) (Figure S5), and tensile tests are conducted on samples with fibers aligned at different angles (Figure 2b). The mechanical properties (e.g., modulus, stretchability, toughness) of the tested substrate varies with the fibers’ alignment angle. In a reticular arrangement (i.e., grid‐like pattern), the fibers may behave like pivots or hinges when subjected to external loads. As the material deforms under stress, the fibers rotate and gradually align to the external loading axis, thereby increasing the composite material overall stiffness. This is because the fibers' intrinsic stiffness is maximized along their length, thus when the fibers are aligned with the loading axis, they resist deformation more effectively. However, in ETATS a suitable trade‐off between modulus and extensibility is needed. Considering this, among the various tested angles (Figure 2b), the highest toughness, still allowing substrate stretchability, is exhibited when the fibers are aligned at 50 degrees. Moreover, adding fibers to the soft skin effectively inhibits crack propagation from the notch cut (Figure 2c) (opposite to the fiber‐free case, see Figure S6), and the compressive modulus increases (Figure S7).

To promote ruggedness against punctures and cuts, we focused on developing a hard discretized layer (similar to the natural stratum corneum in the epidermis) still enabling deformation of the soft layer beneath (similar to the natural dermis). To this aim, an island array is designed and fabricated on the surface of the soft skin.

The hexagonal shape of the islands is found as providing the best trade‐off between stress distribution (affecting structural integrity and load‐bearing behavior) induced by stretching, bending, and torsion, and the variation of the soft polymeric matrix surface area between islands, which must be minimal during deformation for puncture immunity (see Supporting Information and Figure S8). In addition, we evaluated the stress distribution at the base of the rigid islands under stretching with 20% strain. The simulation shows that the maximum von Mises stress for the hexagonal islands is lower than that for the triangular and square islands (Figure S9). In addition, the use of rounded hexagonal islands could potentially enhance stress distribution. The combination of epoxy and TiO_2_ particles is used for creating rigid islands, while nanoclay helps in tuning the viscosity of the starting solution for successful fabrication. Figure 2d shows the fabrication process, in which a curing step by heating triggers robust hydrogen bonding between the oxygen plasma‐treated soft skin and rigid islands [40]. The fabricated hexagonally packed rigid island array (side length of hexagon: 1.7 mm, height of island: 0.8 mm, gap between islands: 1.1 mm) perfectly covers the large‐area of soft skin surface (90 mm × 60 mm × 1 mm) (Figure 2e). Indeed, the absence of nanoclay in the epoxy/TiO_2_ solution leads to undesired void formation (Figure S10). Also, it can be noted how, although using a continuous rigid layer on top of the soft skin would offer the best mechanical protection, it would limit the skin deformation, which would be prone to mechanical failure under stretching (Figure S11).

Results of puncture tests (Ø0.2 mm conical tip) are shown in Figure 2f,g. While islands built without any TiO_2_ particles show mechanical failure at 32N (Figure 2f), testing the inclusion of different concentrations of the latter, as well as curing temperatures, (Figure S12), shows that mechanical robustness can be increased. Results indicate that, indeed, for TiO_2_ concentrations above 10%, and an additional heat treatment at 140°C, puncture resistance is significantly improved (97.5 N) (Figure 2g). The increased hardness of the islands is attributed to the optimal stress distribution enabled by the dispersion of hard TiO_2_ particles within the epoxy matrix (Figure S13), as well as the enhanced crosslinking degree induced by high‐temperature post‐curing [24]. The islands with excellent hardness also contribute to resist mechanical damages caused by, e.g., sharp objects, cutting. To investigate puncture resistance to a needle, force is applied to the soft skin with and without islands using a 25‐gauge hypodermic needle (Figure 2h). The needle easily penetrates the bare soft skin with a force below 2N, while adding islands prevents penetration for externally applied forces up to 13 N. Finally, the resistance to knife cutting is also tested on samples of soft skin with and without islands (Figure 2i). Although the soft skin without islands could be easily penetrated, the soft skin covered with the hexagonal island array remained stable without mechanical failure up to 80 N. This difference in puncture resistance is therefore critical, making the exposed (unprotected) area a key design parameter in the selection of the island geometry (Figure S8).

### 2.2 Armor Skin with Folding under Stretching and Compressing Modes

**FIGURE 3:**
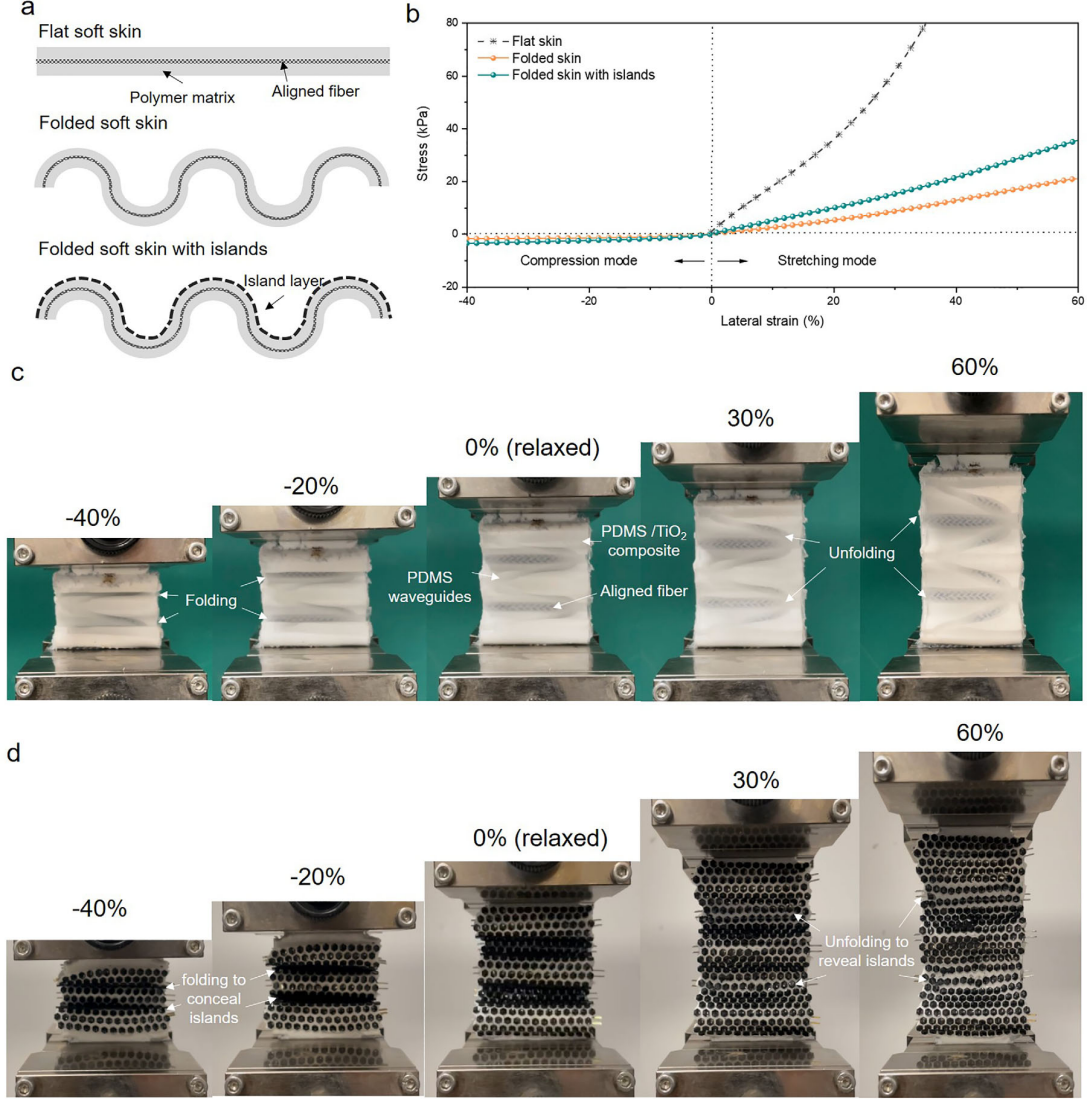
The behavior of ETATS under stretching and compressing modes. a) Schematic illustration of three soft skin configurations. b) Stress versus strain for three soft skin configurations under both stretching and compressing in lateral direction. c,d) Photographs of folded soft skin (c) without and (d) with islands under different strains of ‐40% (compressed), ‐20% (compressed), 0% (relaxed), 30% (stretched), and 60% (stretched).

To enable deformability in the ETATS, while maintaining its armor functionality, the principle of achieving stretching and compressing in lateral direction via folding, similar to the elephant skin, is exploited in the artificial skin. Although the flat soft skin with rigid islands has high stretchability, it is not suitable as stretchable armor because the distance between the islands increases when it is stretched. Additionally, the armor should make conformal contact with the surface of users (human or robots) without hindering their movements, requiring it to have a low modulus and the ability for lateral compressing. Therefore, we introduce a folding structure for obtaining a highly stretchable/compressible soft skin (Figure 3a). To investigate the effect of folding, tension and compression tests in lateral direction are addressed on three configurations of the fiber‐embedded PDMS/TiO_2_ skin: 1) flat soft skin without islands, 2) folded soft skin without islands, and 3) folded soft skin with islands. Here, the folded structure is designed with consideration of the island arrangement, lateral strain and compression should not be affected by the islands (Figure S8), and the folded soft skin is fabricated using 3D printed molds. As shown in Figure 3b, the folded soft skin is highly stretchable and compressible due to its low modulus. Compared with the flat skin, which has a modulus of ≈200 kPa, the folded skin without islands exhibits a significantly reduced modulus of 35.7 kPa in stretching mode and a compressive modulus of 3.46 kPa in compression mode. In contrast, the folded skin with islands shows a slightly higher modulus of 58.3 kPa in stretching mode and a compressive modulus of 7.40 kPa, owing to the incorporation of additional soft skin layer with islands. An additional cyclic stretching and compressing in the lateral direction (‐30%–+30%) was performed. The corresponding stress–strain response was recorded over 1,000 cycles and shows repeatable mechanical behavior without noticeable structural failure across the tested cycles (Figure S14). Figure 3c,d shows the top view of the folded skin without islands and with islands, respectively, at different lateral strains: ‐40% (compressed), ‐20% (compressed), 0% (relaxed), 30% (stretched), and 60% (stretched). Results show that the structure morphologically provides effective protection by folding to conceal and unfolding to reveal the islands as needed (Figure 3d). Under stretching, the exposed PDMS/TiO_2_ area ratio between the islands was calculated (Figure S15). The folded skin with islands showed a lower exposed area ratio than the flat skin with islands by effectively suppressing exposed area expansion.

### 2.3 Armor Skin with Optical Pressure and Strain Sensing

**FIGURE 4:**
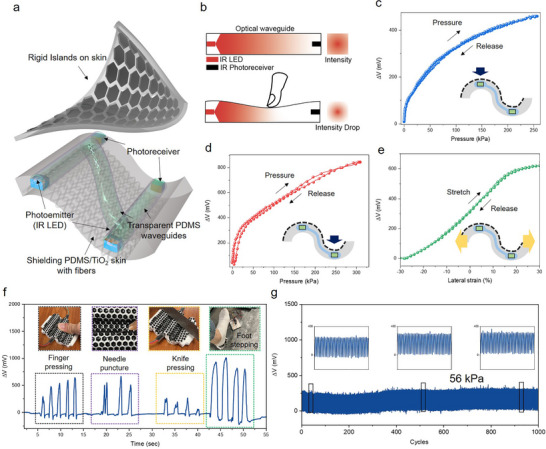
Tactile sensing performance of ETATS. a) Schematic of ETATS. b) Sensing mechanism of optical waveguide‐based tactile sensors. c, d) Relative change in voltage versus pressure of the top (c) and bottom (d) pressure sensors in ETATS. e) Relative change in voltage versus lateral strain of the strain sensor in ETATS. f) change in voltage due to various inputs (finger pressing, needle puncture, knife pressing, and foot stepping). g) Durability tested under 1000 cycles with pressure of 56 kPa (sweep rate: 90 mm min^−1^).

Figure 4a illustrates the schematic representation of the ETATS sensing mechanism, which utilizes both transparent and opaque PDMS structures to enable optical tactile sensing. Optical sensing is selected due to its compatibility with PDMS, ease of signal acquisition, and straightforward operational principles. However, this approach presents certain limitations, including geometric constraints and susceptibility to ambient light interference. To mitigate these issues, a multistep fabrication process is employed, incorporating PDMS with and without reflective TiO_2_ particles. The inclusion of these particles facilitates the formation of a core‐cladding structure, a configuration that has been successfully demonstrated in previous studies [34, 35]. The incorporation of reflective particles also serves as an optical coating, providing additional shielding of the waveguides from external ambient light. Optical waveguide‐based sensors partially covered by the PDMS/TiO_2_ polymer matrix exhibit pronounced light‐induced signal noise (Figure S16). Figure 4b illustrates the basic working principle of optical tactile sensors, where mechanical deformations alter light intensity within the waveguides. Electrical properties of the photoreceiver follow the light intensity change, which can be tracked by acquisition systems. The skin features a “wavy” structure with optical pressure sensors positioned at extremum points (the peaks and valleys of the wave pattern). These waves serve as the primary mechanism for stretching, as deformation primarily affects the wave structure rather than the material itself [41]. During compression in the lateral direction, the wave pattern becomes more pronounced without significantly deforming the material. Consequently, optical pressure sensors based on vertical waveguides are subjected minimal deformation due to the device's wavy structure. This design ensures that pressure sensors remain unaffected by in‐plane uniaxial mechanical deformation, providing accurate pressure measurements. Finite element (FE) simulations were performed to analyze the stress and deformation distribution under tensile strain up to 30% (Figure S17). The simulations indicate that the deformation of the optical sensing elements remains below 10% for the top pressure sensors and below 20% for the bottom pressure sensors. These deformation levels do not affect the sensor baseline, as further demonstrated experimentally in Section 2.4. To enhance functionality, diagonal waveguides were introduced, serving as optical strain sensors. Unlike pressure sensors, these waveguides are positioned away from extremum points, causing them to undergo greater deformations during linear stretching and compression in the lateral direction, making them ideal for lateral strain sensing. The top and bottom pressure sensors respond differently to external pressure due to structural non‐uniformity (Figure 4c,d). The top sensor is less sensitive because of the cavity beneath it, allowing for vertical displacement under pressure. In contrast, the bottom sensor lacks this flexibility. The strain sensors (Figure 4e) exhibited an almost linear response and remained unaffected by pressure, despite using the same electrical components as the pressure sensors. The algorithms for distinguishing between these two types of deformations will be discussed in the next section. The skin responded in real time to various inputs (finger, needle, knife, and foot), effectively detecting and withstanding forces of different magnitudes while providing complete protection without physical damage (Figure 4f). This demonstration suggests that the ETATS can safely protect and detect stimuli even under harsh conditions. To assess durability, tests for 1000 loading‐unloading cycles with a pressure of 56 kPa at a sweep rate of 90 mm min^−1^ confirm consistent real‐time response (Figure 4g). In addition, the skin shows response and recovery time of 90 and 50 ms, respectively (Figure S18).

### 2.4 Large Area Sensing in Real‐Time

**FIGURE 5:**
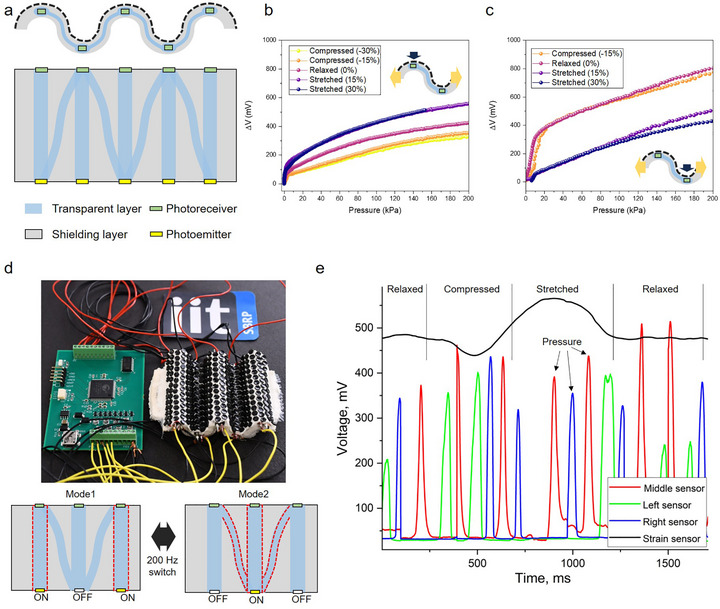
Large‐area sensing of ETATS. a) Schematic of ETATS including five optical pressure sensors and four optical strain sensors. b) Voltage versus pressure for the top waveguide for different states of the skin. c) Voltage versus pressure for the bottom waveguide for different states of the skin. d) Photograph of ETATS connected with a custom‐designed PCB. To enable large‐area sensing, the photoemitters operate with high‐speed switching. e) Real time monitoring of the sensor array.

Using the principles described in the previous section, we developed the ETATS for large‐area sensing. The principles and algorithms discussed in this section can be extended to support a greater number of sensors. The developed ETATS includes five optical pressure sensors and four optical strain sensors, as shown in Figure 5a. As mentioned earlier, the device can detect two types of deformation: uniaxial stretching/compression in lateral direction and pressure. Stretching or compression in lateral direction does not significantly affect the pressure sensors; it only alters their sensitivity, as demonstrated in Figure 5b,c. When the device is stretched, the upper pressure sensors become more sensitive, while compression reduces their sensitivity. The lower pressure sensors exhibit the opposite behavior—their sensitivity decreases under stretching. These sensitivity changes result from structural modifications of the device during deformation. In future studies, this issue can be addressed by applying a calibration matrix based on the stretched state of the skin. This approach would require separate calibration curves for different deformation states, allowing the pressure signal to be compensated according to the simultaneously measured strain. However, despite these structural changes, the graphs show that even at 30% elongation, the baseline values of the pressure sensors remain unchanged. One of the key innovations of this research is the development of a device that uses the same set of electrical components to detect different types of mechanical deformation. The algorithm, as well as the software and the hardware were designed specifically for the sensing mechanism based on the diagonal waveguide structure. The pressure sensor consists of a photoemitter, a photoreceiver, and a transparent vertical waveguide, as shown in Figure 5d. The photoemitter and photoreceiver can be selectively activated or deactivated via a custom‐designed PCB (Figure S19). The PCB contains transistors that activate and deactivate the photoemitters. Depending on the photoemitter state, data from the photoreceivers is analyzed differently. The strain sensors are formed by diagonal waveguides that connect the photoemitter of one pressure sensor to the photoreceiver of another, as illustrated in Figure 5d. By sequentially switching the photoemitters on and off, distinct signals can be captured from individual waveguides. For example, when the central photoemitter is deactivated (Mode 1), pressure is measured through the side waveguide‐based sensors. Conversely, when the side photoemitters are deactivated (Mode 2), the central vertical waveguide‐based sensor records pressure data, while the diagonal waveguide‐based sensors provide information about strain or compression in lateral direction. The device switches between these modes at a frequency of 200 Hz, enabling simultaneous and differentiated detection of pressure and strain over a large sensing area. Although it is possible to measure strain for each diagonal waveguide‐based sensor, in this case demonstrated strain is a sum of voltage change for all strain sensors. For larger‐area skins, the number of operating modes can be increased according to the number of strain regions to be measured. In the current architecture, pressure sensing is performed in a single mode (all pressure channels read simultaneously), whereas strain sensing requires selective deactivation of specific emitters to isolate the strain channels. As the number of strain regions (and thus strain‐specific modes) increases, the time‐multiplexing overhead grows and the effective update rate of the full device decreases, unless parallel readout and/or parallel signal processing is implemented. In the case of susceptibility to external ambient light, an additional measurement mode can be introduced. In this mode, all photoemitters are turned off so that the ambient light level is recorded. This background signal can then be subtracted from the signals obtained in Mode 1 and Mode 2, enabling compensation for ambient light interference.

As shown in Figure 5e and demonstrated earlier, pressure sensing (represented by the red, green, and blue curves) is not influenced by strain. However, the strain signal (black curve) is partially affected by applied pressure (small voltage increase in relaxed mode). This effect arises because the pressure alters the overall shape of the device, leading to cross‐sensitivity. In future work, this coupling will be addressed by applying machine learning techniques and by optimizing the structural design of the strain sensors.

## 3 Discussion

In this work, we presented the development of the elephant trunk‐inspired armor skin with tactile sensing (ETATS). The design incorporates a hierarchical structure inspired by the anatomical features of the natural model, addressing three key aspects: mechanical robustness, deformability, and tactile sensing.

Regarding mechanical robustness, the internal structure of natural elephant trunk skin is composed of aligned collagen fibers that are both robust and deformable, while the outermost layer is formed by relatively stiff discrete islands. Considering these characteristics, the soft polymer matrix of ETATS is embedded with aligned plastic fibers that mimic the function of collagen fibers, and rigid hexagonal island array is integrated on the top surface of the skin. The mechanical properties of the soft polymer matrix can vary significantly depending on the alignment angle of the embedded fibers, with 50° orientation enhancing mechanical properties such as toughness, stretchability, and crack resistance. Additionally, the rigid hexagonal island array exhibits a 2.75‐fold increase in puncture resistance compared to natural elephant trunk skin. The PDMS/TiO_2_/nanoclay composite‐based islands arranged at regular intervals provide high deformability while effectively protecting the underlying soft skin from mechanical damage. Nevertheless, the soft skin between the islands still remains exposed and is susceptible to expand under various deformation modes. This effect is partially mitigated by the stress distribution in the wrinkled structure, so the exposed area starts to increase only at higher strains (Figure S15).

Regarding deformability, the thick skin (≈15 mm) of natural elephant trunk exhibits both stretchability and compressibility of 30% through its wrinkle/fold structures. While the wrinkle/fold morphology differs by anatomical location of the elephant trunk [17], ETATS incorporates a simplified wavy(fold) structure. This configuration allows for up to 60% tensile strain and 40% compressive strain in lateral direction. Moreover, when stretched, the folded structures open exposing hidden islands, which contribute to effective protection. To the best of our knowledge, this is the first demonstration of a highly stretchable and compressible armor architecture beyond previously reported flexible or low‐stretchable (<20% strain) armor systems [20, 21, 22, 23, 24, 25]. Although our folding structure is currently limited to deforming in one direction, we expect that a multidirectional folding pattern could enable more complex motions, such as multiaxial stretching, twisting, and curling, similar to those of natural elephant trunk.

Regarding tactile sensing, the elephant trunk skin contains distributed mechanoreceptors that allow sensing external applied forces and induced strains. We propose a new architecture for large‐area tactile sensing, based on a folded surface design. Optical pressure sensors were placed at the extremum points of the structure, while optical strain sensors connect these points. This configuration enables simultaneous measurement of both pressure and strain using the same hardware components, made possible by our custom sensing algorithms. Finally, we demonstrate ETATS capable of differentiating various tactile stimuli, validating its potential for practical applications. Similar to the principle of stretchable armor, the fold structures unfold upon stretching, revealing hidden optical waveguide‐based sensors, which contributes to effective tactile sensing. Furthermore, by incorporating machine learning algorithms into our signal processing methods, we expect to minimize signal crosstalk in the sensing data and achieve more reliable artificial skin.

While the current hierarchical architecture is fabricated using a multi‐step molding process, adopting advanced 3D printing techniques [42, 43, 44] could significantly streamline the fabrication workflow and enable further scaling down. Moreover, although optical waveguide‐based sensors are utilized to ensure long‐term reliability, the integration of alternative soft sensors, including piezoresistive [45] and capacitive sensors [46], could facilitate the development of compact, high‐resolution tactile armor skins.

Various soft sensor‐embedded robotic skins have been integrated with both rigid and soft robots [44, 47, 48, 49, 50, 51, 52]. These robotic skins have successfully demonstrated highly sensitive tactile sensing. However, limitations in deformability and insufficient mechanical robustness may hinder their practical applications in highly deformable soft robots. Therefore, ETATS can benefit both humans and robots, and is especially valuable for soft robotics.

## 4 Materials and Methods

### 4.1 Preparation of 3D Printed Molds

As shown in Figure S3a, Rigid 2000 (Formlabs, USA) molds were manufactured using stereolithography (SLA) type 3D printer (Form 3, Formlabs). Chemical treatment of the molds for peeling polymer substrates easily was conducted. The 3D printed molds were treated with O_2_ plasma (Power: 100 W, Duration: 1 min). After that, the treated molds were placed in a sealed beaker with parafilm and then exposed to the vapor of Trichloro(1H,1H,2H,2H‐perfluorooctyl)silane (Sigma‐Aldrich) on the hot plate at 70°C for 30 min [48]. To remove residual chemicals, the molds were cleaned with ethanol.

### 4.2 Fabrication of Wrinkled PDMS/TiO_2_ Composite with Aligned Fibers

The PET fibers (PET Braided Cable Sleeving, 30 mm diameter, RS PRO) were placed on 3D printed mold with specific angles. At this moment, the fibers were fixed onto the mold using a heating gun to create a wrinkled shape. After that, it was covered with another 3D printed mold and pressed using clamps (Figure S3b). To prepare prepolymer PDMS/TiO_2_ composite solution, prepolymer PDMS solution (SYLGARD 184, the base and curing agent in a weight ratio of 30:1) and TiO_2_ powder (<10 µm, Sigma‐Aldrich) were mixed in a weight ratio of 50:1 with vortex mixer (2000 rpm, 1 min). The prepolymer PDMS/TiO_2_ solution was poured into the holes of molds. The degassing chamber helps ensure that the solution completely fills the molds. Finally, it was cured in the oven at 80°C for 2 h. Then, cured PDMS/TiO_2_ with fibers was peeled off from the molds (Figure S3c).

### 4.3 Fabrication of Optical Waveguide‐Based Sensors

To form sensing parts in wrinkled PDMS/TiO_2_ with fibers, five photoreceivers (TEMT7100×01) and five photoemitters (infrared LEDs, VSMY1850) were placed on the end of each waveguide (Figure S3d). After that, the prepolymer PDMS solution (the base and curing agent in a weight ratio of 30:1) was poured into the empty spaces to form transparent waveguides. It was cured in the oven at 80°C for 2 h.

### 4.4 Fabrication of Hexagonal Epoxy/TiO_2_/Nanoclay Island Array

To fabricate supporting (base) layer, the prepolymer PDMS/TiO_2_ solution was prepared (the composition is same with previous step). The PDMS/TiO_2_ membrane (thickness: 1.25 mm) was manufactured using bar‐coating machine. After that, the membrane was treated with O_2_ plasma for robust bonding between PDMS/TiO_2_ and epoxy‐based island array. Meanwhile, a stretchable and adhesive mask for island array was prepared by pouring Ecoflex‐10 (Smooth‐on, the base and curing agent in a weight ratio of 1:1) solution into 3D printed mold. To prepare the solution for the islands, prepolymer epoxy solution (Epoxycast, Smooth‐on) was used, the base and curing agent in a weight ratio of 10:3), TiO_2_ powder, and Nanoclay (Sigma‐Aldrich) were mixed in a weight ratio of 20:2:1 with vortex mixer (2000 rpm, 1 min). Here, small amount of black ink (Smooth‐one) was added. The epoxy/TiO_2_/nanoclay composite solution was blade coated on the PDMS/TiO_2_ layer with Ecoflex‐10 mask (Figure S3e). It was cured in the oven at 80°C for 12 h. For additional heat treatment, it was heated in the oven at 140°C for 1 h.

### 4.5 Integration of Folded PDMS/TiO_2_ Skin and Island Layer

To make adhesive layer, PDMS (the base and curing agent in a weight ratio of 10:1) and TiO_2_ powder were mixed in a weight ratio of 50:1. The adhesive was applied to each of the two layers (folded PDMS/TiO_2_ skin and island layer), and after attaching them, pressure of 100gf was applied in the oven at 70°C for 4 h (Figure S3f).

### 4.6 Mechanical Testing

Strain tests and puncture resistance tests were conducted using a universal testing machine (Zwick Roell, Ulm, Germany) with a custom FDM‐printed test fixtures. In the puncture and cut test, soft skin with and without islands was pressed using conical sharp (diameter: 0.2 mm), 25‐gauge hypodermic needle, and knife. The motor speed was 10 mm min^−1^. Pressure tests were conducted by using a micrometric servo‐controlled linear stage M‐111.1DG (Physik Instrumente, Karlsruhe, Germany) with 3D printed additional parts. Strain was applied by using a micrometric servo‐controlled linear stage M‐414.3PD with 3D printed additional parts.

### 4.7 Elephant Skin Specimen Provenance and Preparation

The photographs in Figure 1 were taken from an adult African elephant housed at ZooSafari (Fasano, Italy). The specimens presented in Figure S1 originate from a different adult African elephant that died of natural causes at Zurich Zoo (Switzerland). The trunk was acquired by the University of Geneva for anatomical studies, where initial sectioning was performed. Segments approximately 200 mm in length, collected from different regions of the trunk, were subsequently provided to the Istituto Italiano di Tecnologia for further analysis. Samples were stored at −28°C until testing. All procedures complied with international regulations governing the handling and transport of protected species. Re‐export and import permits were issued by the Swiss and Italian authorities in accordance with the Convention on International Trade in Endangered Species of Wild Fauna and Flora (CITES). Figure S1a shows a proximal–dorsal trunk region with the characteristic folded skin structure, as received from the University of Geneva. The specimens used in Figures S1c and S2a were further sectioned from the larger frozen samples using a surgical blade to ensure precise linear cuts, yielding specimens detached from underlying muscles and with approximate dimensions of 10 mm × 60 mm.

## Funding

European Union's Horizon 2020 program under grant agreement n. 863212 project PROBOSCIS, Italian Ministry of Foreign Affairs and International Cooperation, project DESTRO grant n. PGR02061.

## Conflicts of Interest

The authors declare no conflicts of interest.

## Supporting information




**Supporting File**: advs74963‐sup‐0001‐SuppMat.docx.

## Data Availability

The data that support the findings of this study are available from the corresponding author upon reasonable request.
