# Supplementary material for: The Elephant Trunk Skin Inspires a Highly Sensitive and Deformable, Yet Robust, Armor Skin
Source: Adv Sci (Weinh). 2026 Mar 25;13(32):e74963. doi: 10.1002/advs.74963 (PMC13252638; doi:10.1002/advs.74963)
Supplement: Supplementary file 1 — Supporting File: advs74963‐sup‐0001‐SuppMat.docx. [file ADVS-13-e74963-s001.docx]

Supporting Information

**The elephant trunk skin inspires a highly sensitive and deformable, yet robust, armor skin**

*Jun Chang Yang^1†^, Petr Trunin^1,2†^, Behnam Kamare^1,3^, and Lucia Beccai^1^**

^†^J.C.Y. and P.T. contributed equally to this work

[1] J. C. Yang, P. Trunin, B. Kamare, L. Beccai

Soft BioRobotics Perception Lab, Istituto Italiano di Tecnologia, 16163 Genova, GE, Italy.

[2] P. Trunin

The Open University Affiliated Research Centre at Istituto Italianodi Tecnologia (ARC@IIT), Istituto Italiano di Tecnologia, Genova, Italy

[3] B. Kamare

The BioRobotics Institute, Scuola Superiore Sant’Anna, 56025 Pontedera, Italy

[*] Corresponding Author E-mail: [Lucia.Beccai@iit.it](mailto:Lucia.Beccai@iit.it) (L.B.)


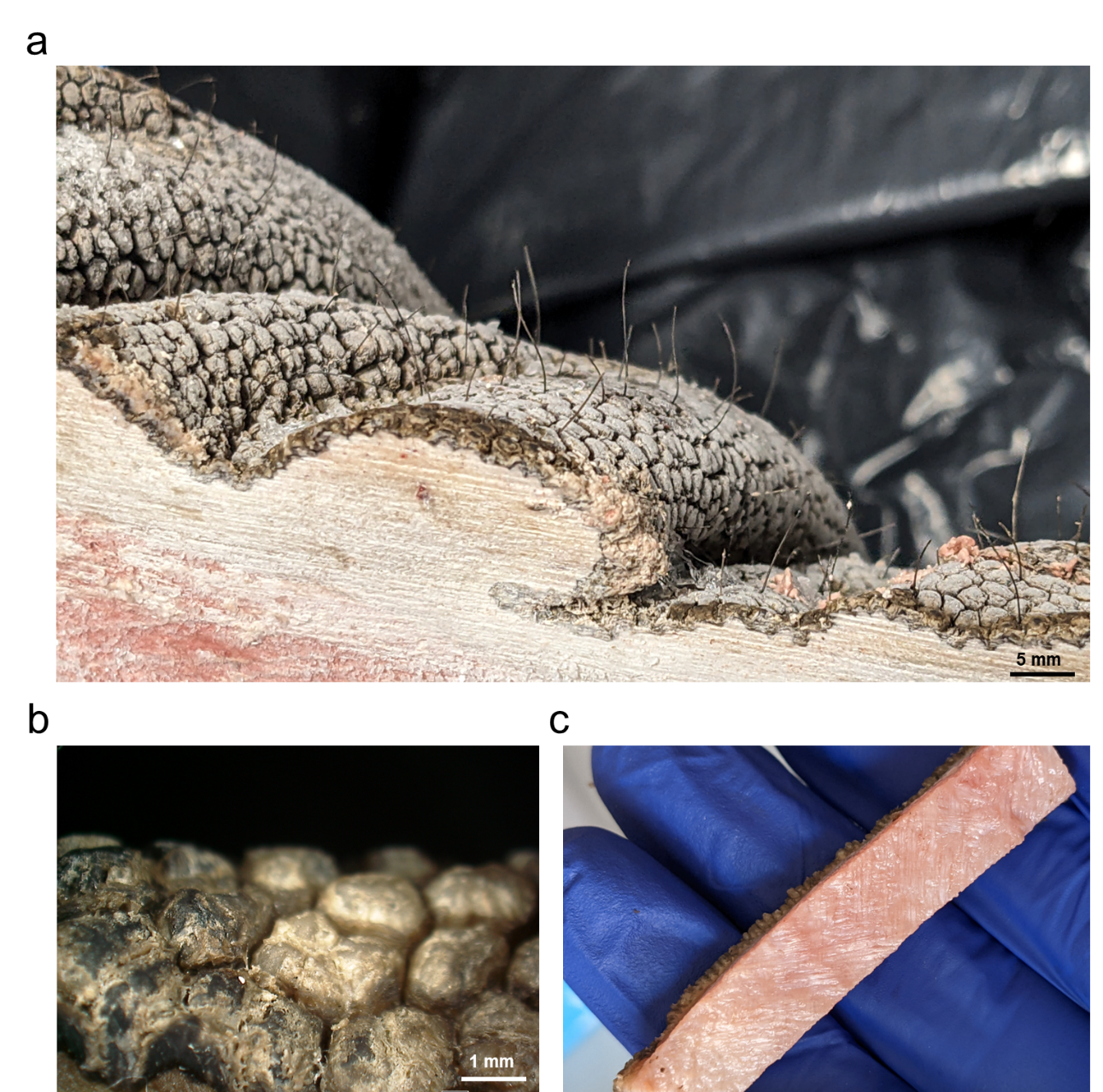


**Figure S1**. Structure of elephant trunk skin from an adult African elephant, all sampled from the proximal–dorsal region (1 m from the tip). a) Folded skin morphology in a cross-section of a frozen sample. b) Island-like architecture of the stratum corneum. c) Dermal-side view of a specimen (width=10 mm) showing the reticular network of collagen fibre bundles with two dominant orientations.


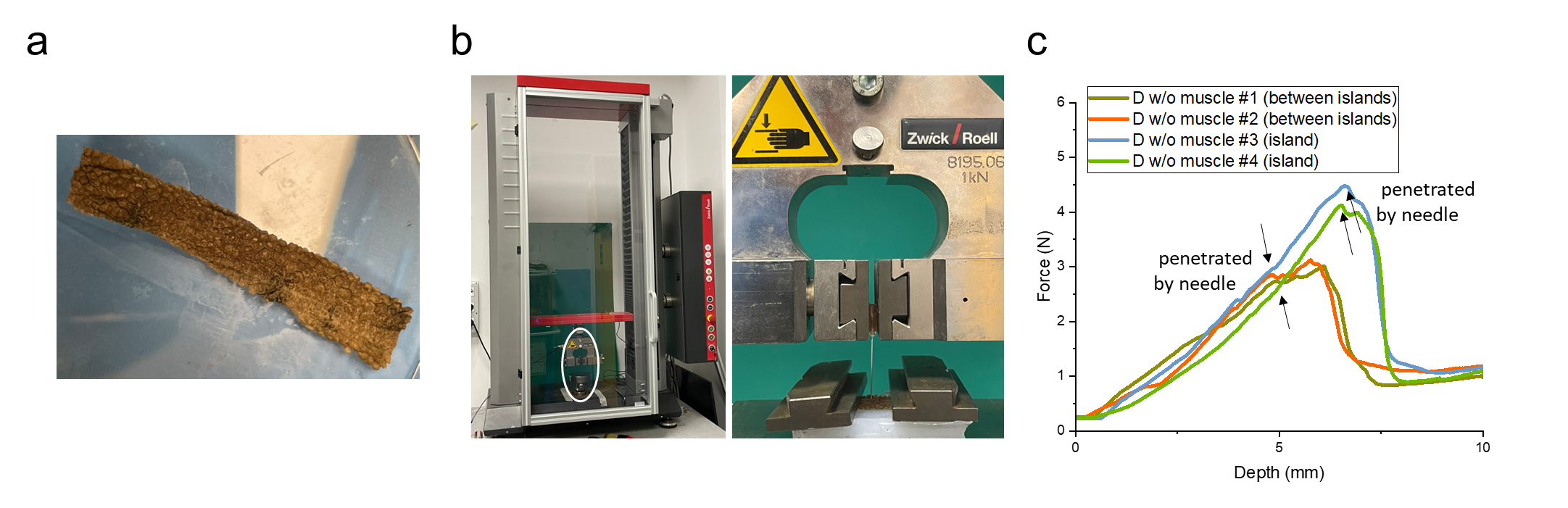


**Figure S2**. Puncture test on elephant trunk skin. a) Elephant skin sample from medial dorsal region (49 cm from tip) of trunk (10 × 60 mm) was detached from underlying muscles and was used for puncture tests. b) The photographs show elephant trunk skin penetrated using 25-gauge needle. Puncture test using 25-gauge hypodermic needle (diameter: 0.5 mm). The motor speed is 10 mm/min. c) Force–depth curve showing higher puncture resistance on the islands than between them.


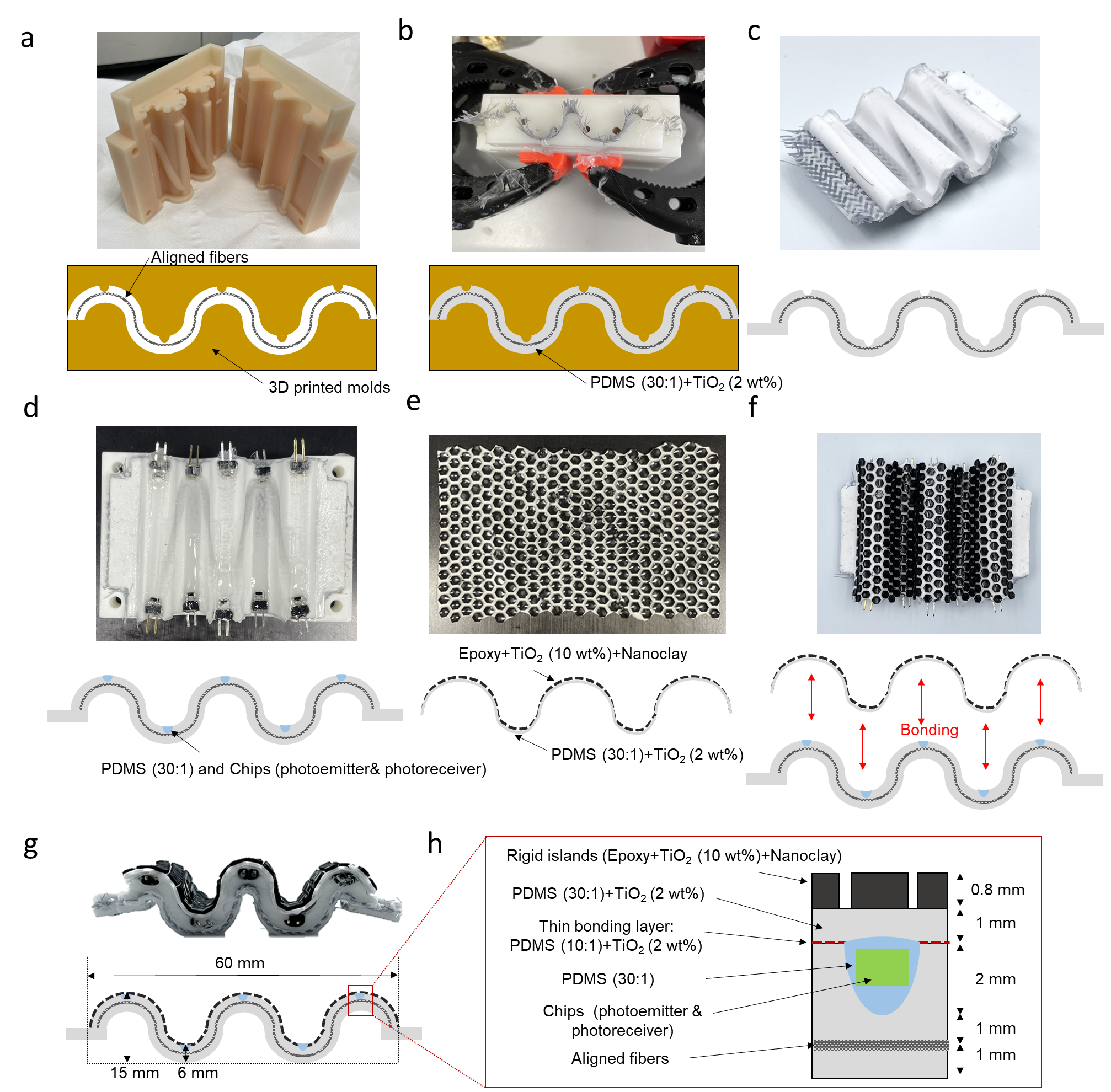


**Figure S3**. Fabrication process and schematic illustration of ETATS. a) Preparation of 3D printed molds with aligned PET fibers. b) Pouring of PDMS/TiO_2_ composite solution into the prepared molds. c) After curing, peeling the wrinkled substrate with aligned fibers from the molds. d) Pouring PDMS into the cavity of the wrinkled substrate and fixing optical chips (photoemitter and photoreceiver). e) Fabrication of an epoxy/TiO_2_/nanoclay composite island array on the PDMS/TiO_2_ composite thin film. The rigid island array is fabricated using the doctor-blading technique. f) Bonding the wrinkled substrate (d) with island array layer (e) using pre-cured PDMS/TiO_2_ composite. g) Fully fabricated ETATS. h) The cross-sectional view of the ETATS.


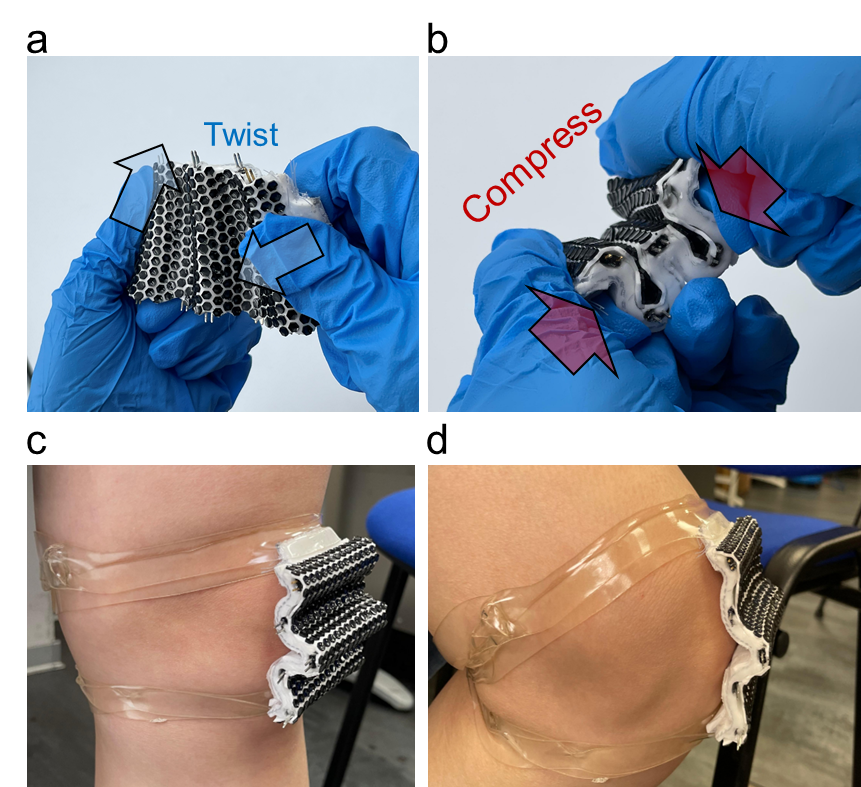


**Figure S4**. Photographs of ETATS (a) under twisting, (b) under compression, (c,d) attached to a knee using 3M tape, showing high deformability during knee bending motion.


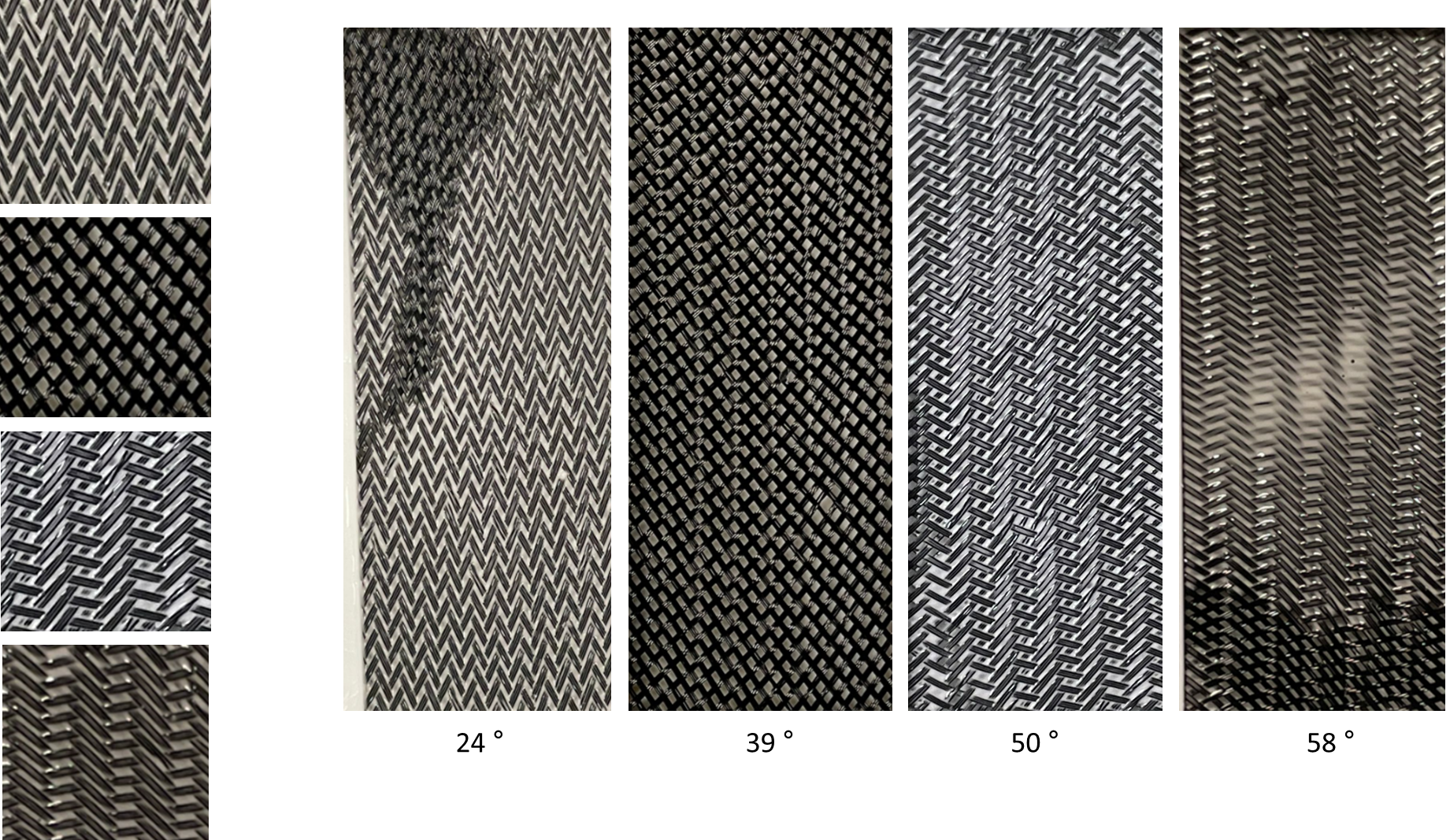


**Figure S5**. Photographs of aligned PET fibers with different angles (24 °, 39 °, 50 °, 58 °) embedded in PDMS/TiO_2_ composite matrix.


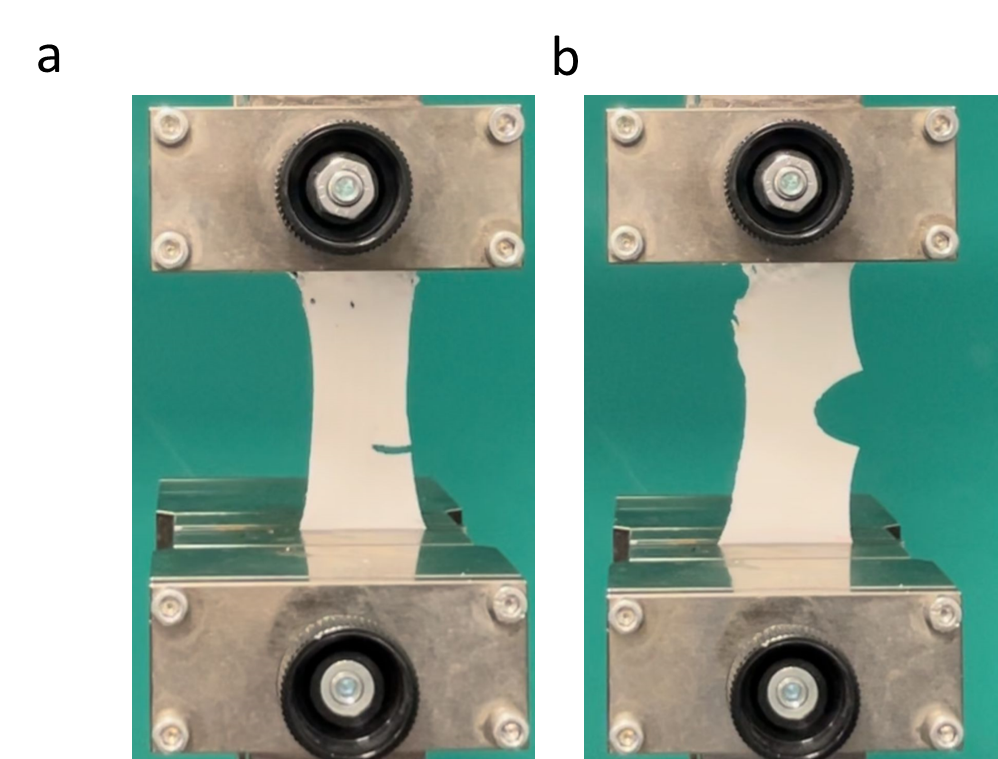


**Figure S6**. Tensile test of PDMS/TiO_2_ composite with notch. The pre-existing notch cut grows rapidly, leading to mechanical failure.


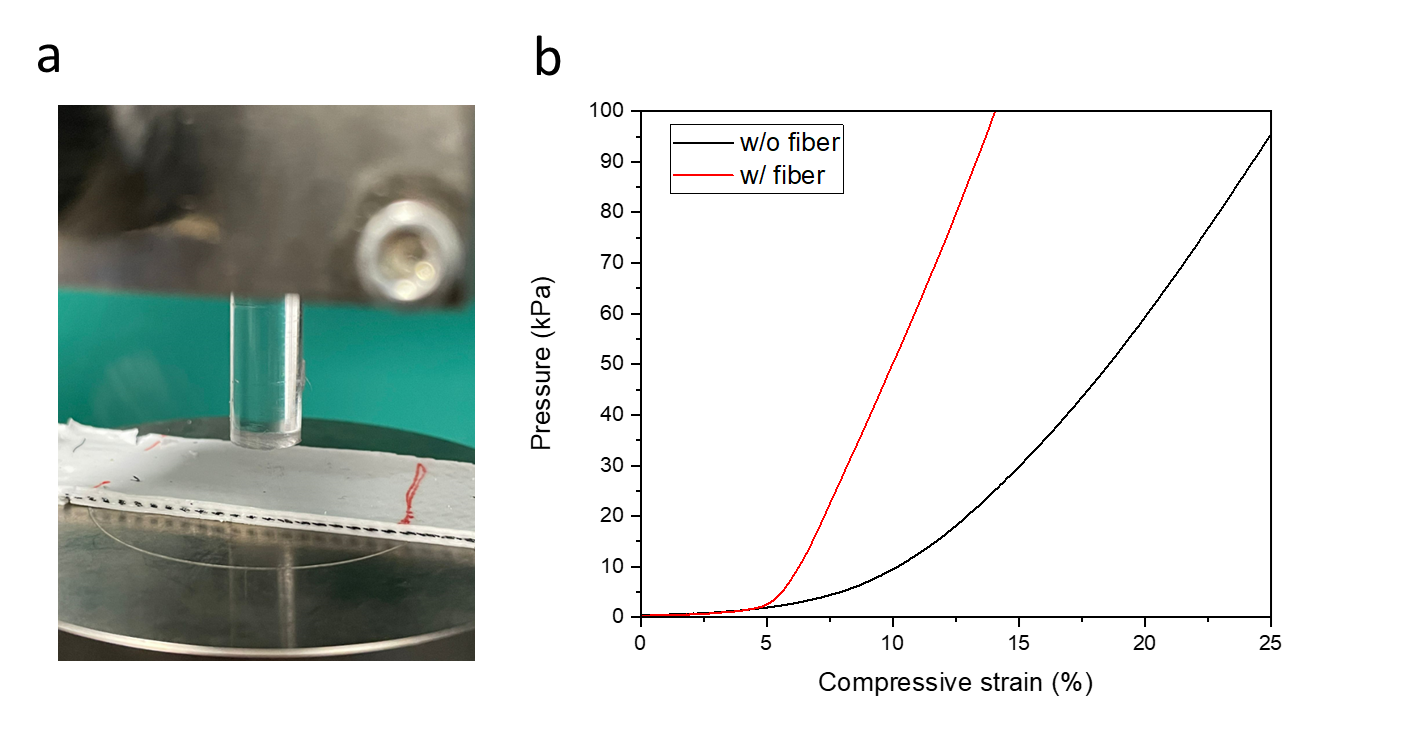


**Figure S7**. a) Photograph of compression test for PDMS/TiO_2_ with aligned fibers (angle:50 °) b) Pressure versus compressive strain curve of PDMS/TiO_2_ without and with aligned fibers. For the PDMS/TiO_2_ without aligned fibers, the compressive modulus was 28.8 kPa (compressive strain: 0~5%), 146.9 kPa (compressive strain: 5~10%), and 502.5 kPa (compressive strain: 10~20%). On the other hand, the PDMS/TiO_2_ with aligned fibers exhibited higher compressive modulus of 31.3 kPa (compressive strain: 0~5%) and 1,114 kPa (compressive strain: 5~15%). We observed that there was no significant difference in compressive modulus up to 5%, but beyond 5%, the modulus approximately doubled due to the effect of the aligned fibers.


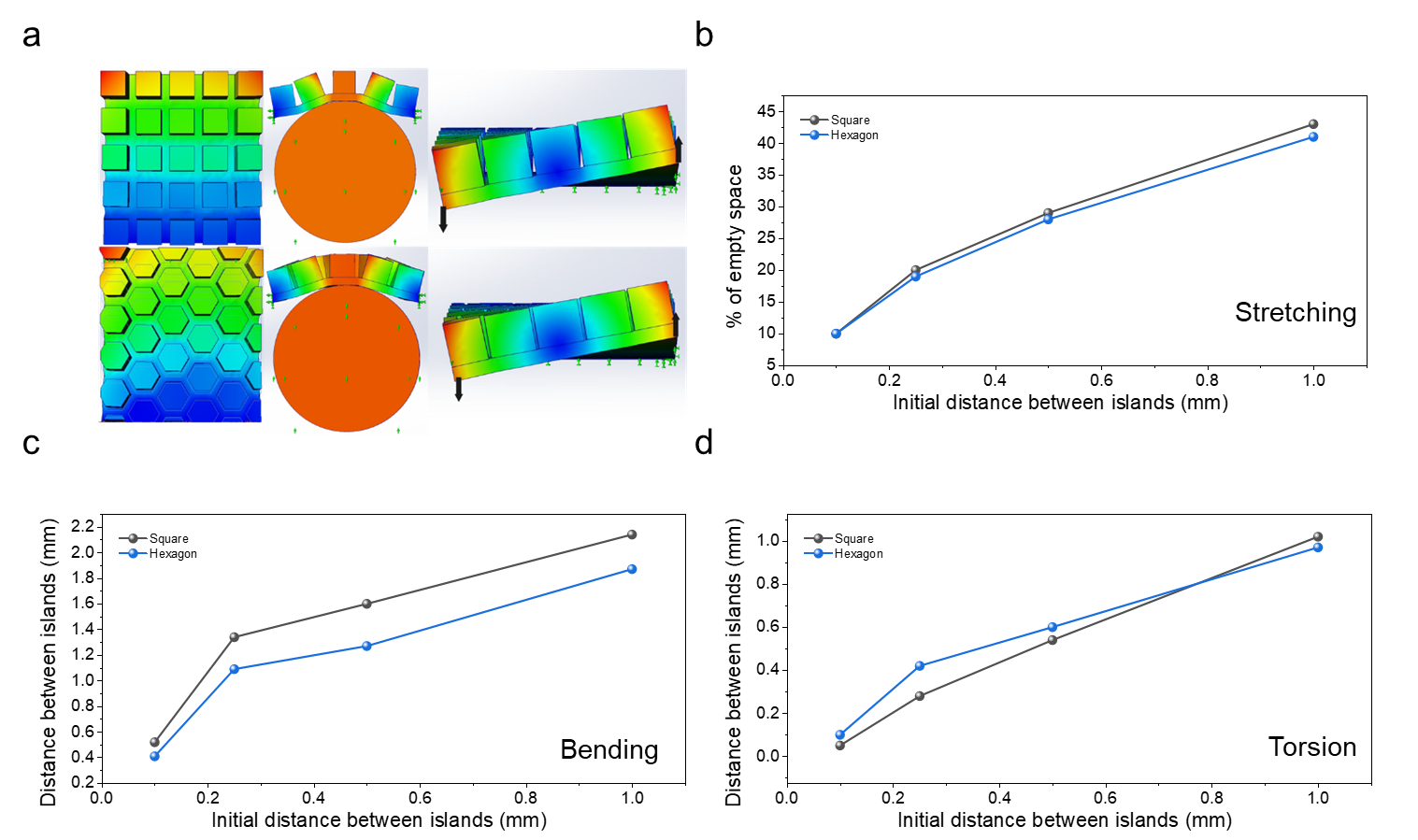


**Figure S8**. Comparison of exposed skin areas between square and hexagonal islands under various deformation modes by a finite element (FE) simulation. a) The FE simulation images showing soft skin with square and hexagonal islands under uniaxial stretching (left), outward bending (middle), and torsion (right). The selection of island shapes was based on fundamental geometric figures capable of tessellating a plane. A 15 mm × 15 mm region was then selected and tessellated with islands of 9 mm^2^ area each. To account for manufacturing constraints that make continuous tiling unfeasible, four samples were tested with varying initial distances between the islands (0.1, 0.25, 0.5, and 1 mm). b-d) Variation in distance between hexagonal and square islands under stretching at 10% strain (b), bending (c), and torsion (d). In conclusion, the hexagonal tessellation demonstrated smaller variations in the uncovered surface area compared to the square tessellation under various deformation modes. To determine the optimal island shape, FE simulations were conducted in SolidWorks. The substrate of the region was modeled with the modulus of PDMS (3 MPa), while the islands were modeled with the modulus of epoxy (3 GPa).


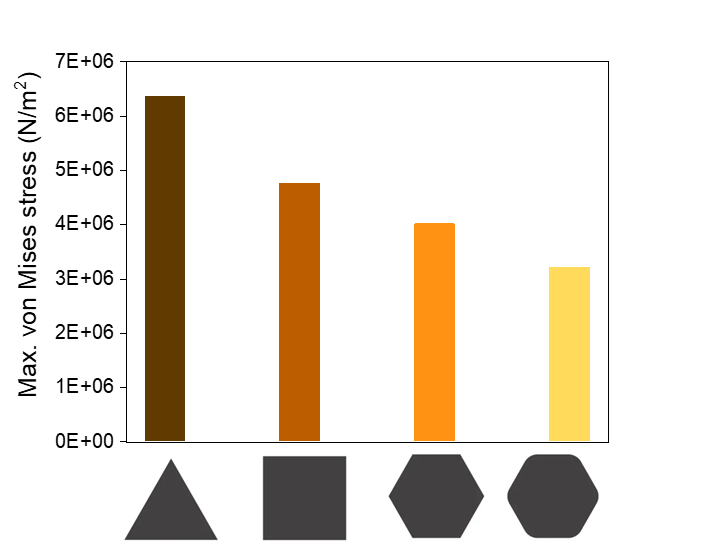


**Figure S9**. Maximum von Mises stress on the base of the triangular, square, hexagonal and rounded hexagonal islands from a finite element (FE) simulation using Solidworks. The polymer matrix with islands was stretched with 20% strain.


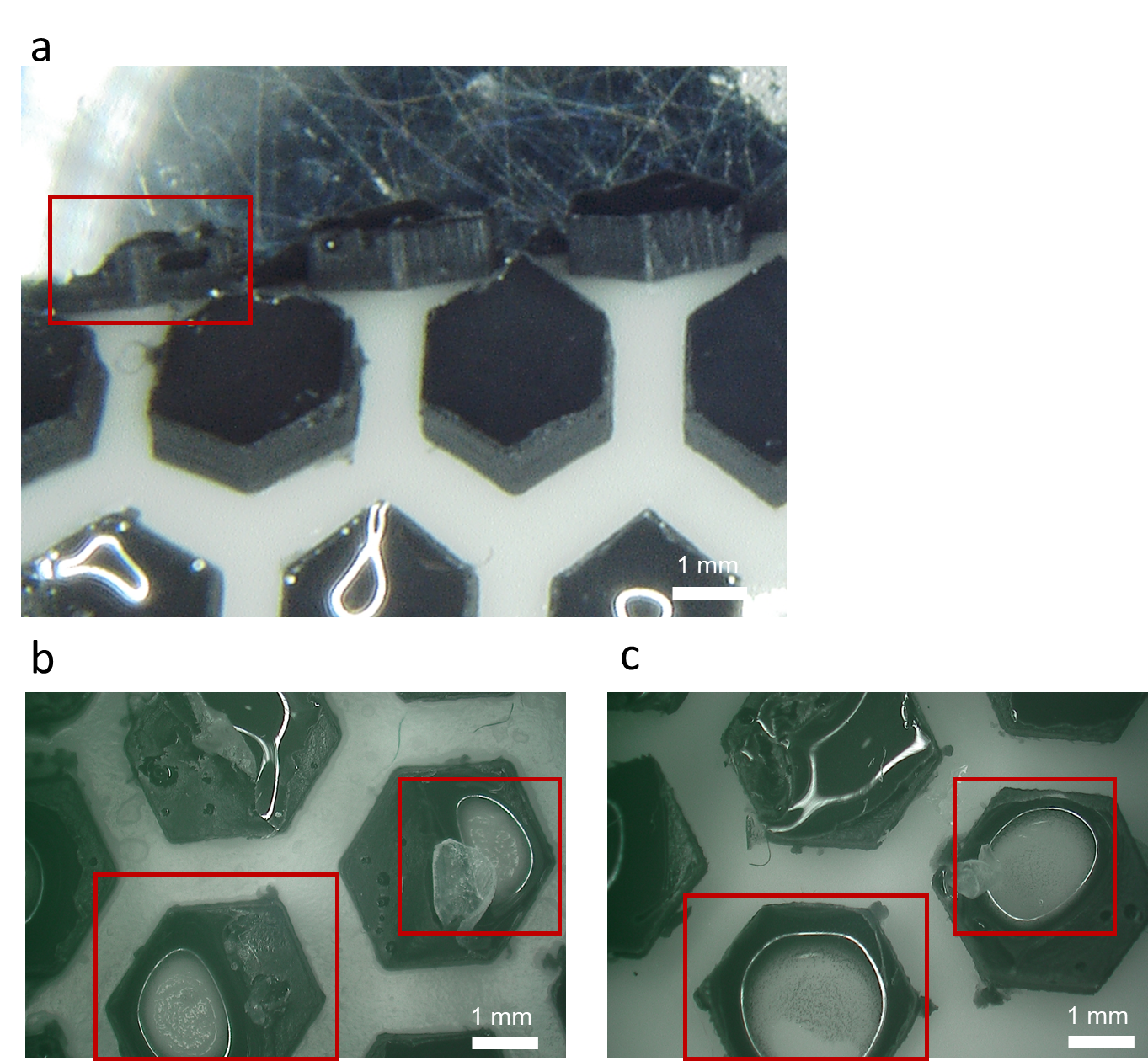


**Figure S10**. Morphologies of the epoxy/TiO_2_ composite island array without nanoclay on the PDMS/TiO_2_ substrate. During the doctor blade process, the epoxy/TiO_2_ prepolymer solution failed to completely fill the open areas of the mask, resulting in holes (red box) in the islands.
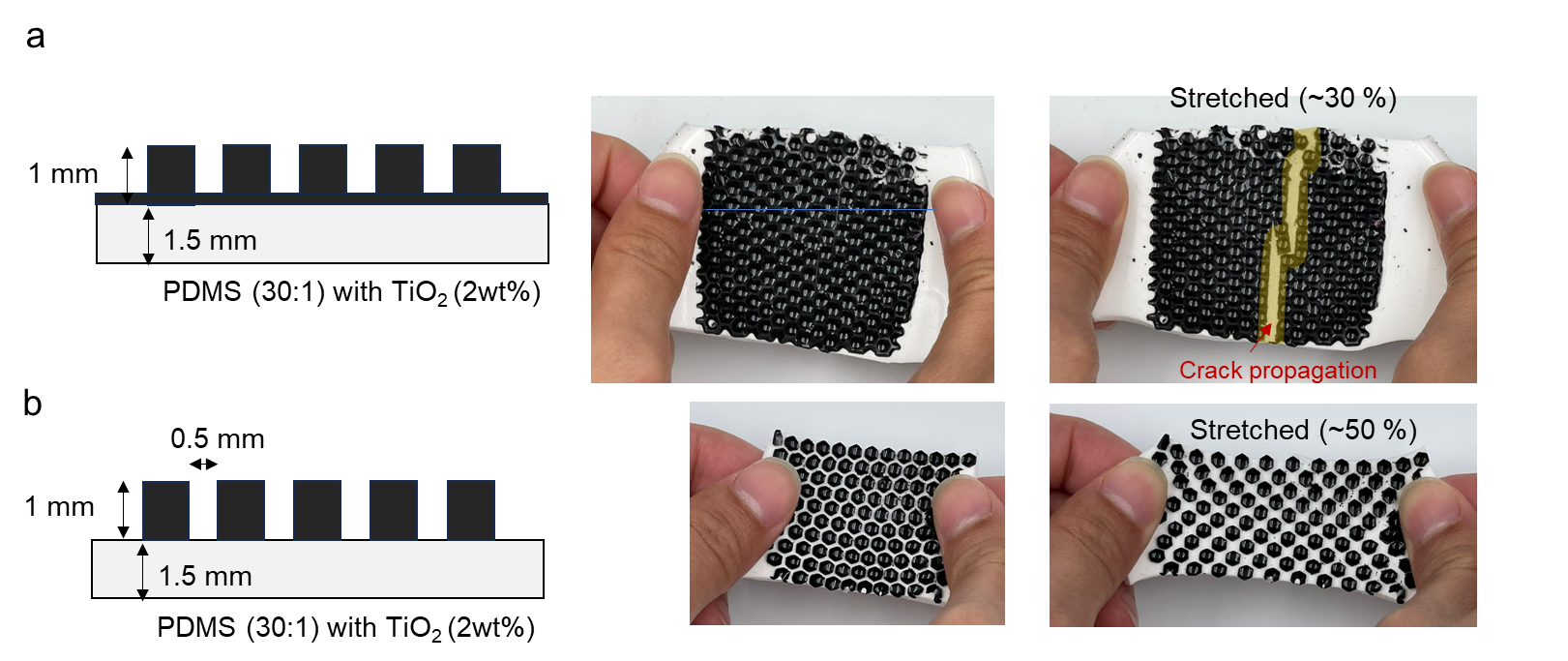


**Figure S11**. Stretchability of PDMS/TiO_2_ substrate with connected and disconnected island array. a) For the PDMS/TiO_2_ substrate with a connected island array, stretchability is less than 30%. b) For the PDMS/TiO_2_ substrate with a disconnected island array, stretchability is higher than 50%.


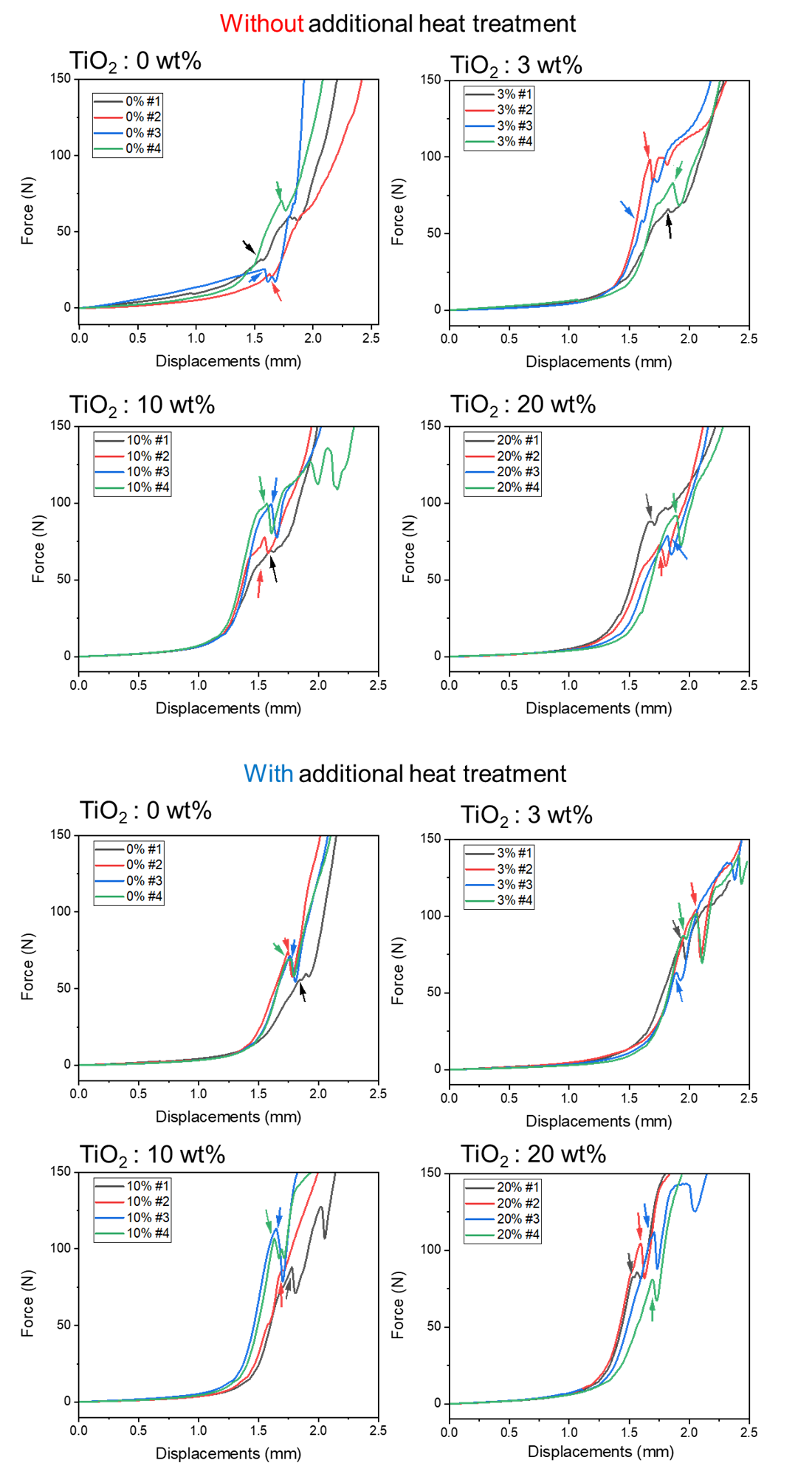


**Figure S12**. Force versus displacement for epoxy/TiO_2_/Nanoclay islands with the different TiO_2_ concentration and post-curing during puncture test. The mean and standard deviation were calculated from four samples under each condition. The arrows indicate the points where mechanical failure occurred.


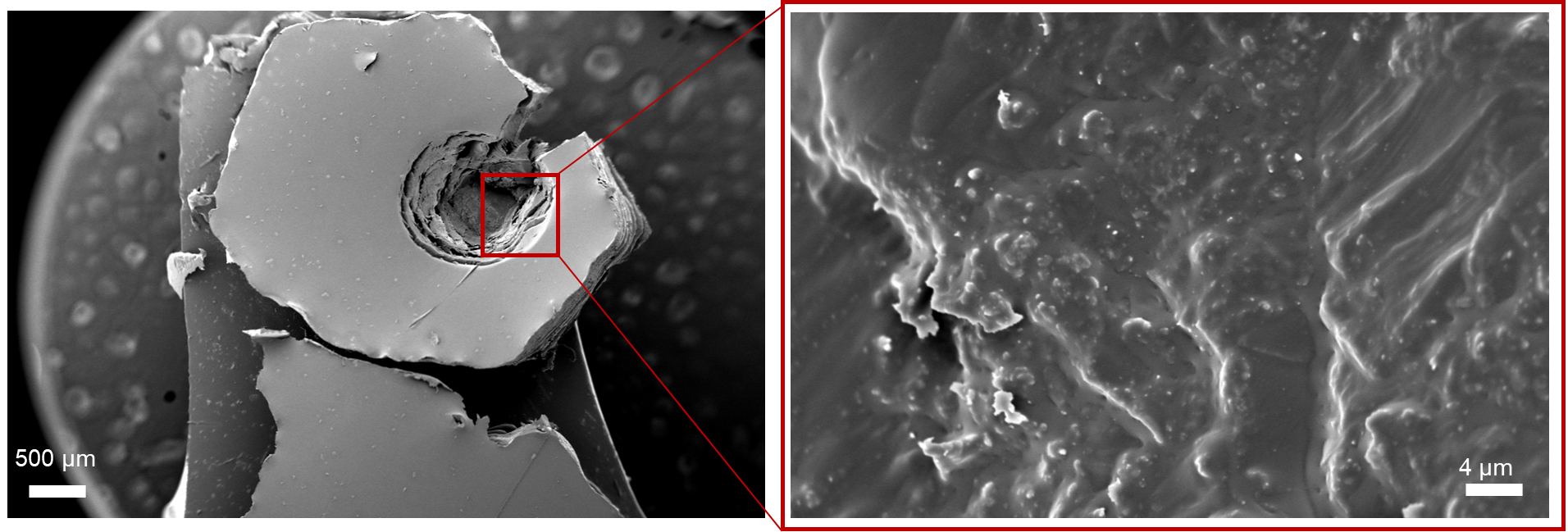


**Figure S13**. SEM image of epoxy/TiO_2_/nanoclay composite islands after puncture test. The TiO_2_ particles reinforce hardness of epoxy islands.


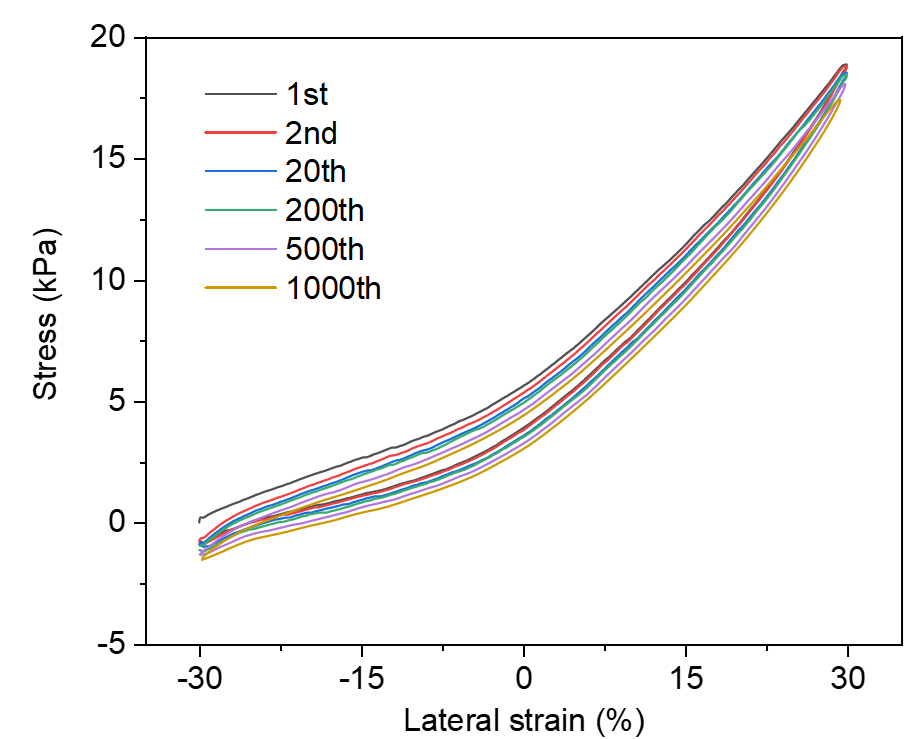


**Figure S14**. Strain-stress curve of ETATS under repeated stretching (+30%) and compressing (-30%) for 1,000 cycles. The nearly overlapping strain-stress (S-S) loops indicate that the ETATS maintains mechanical stability without structural failure during repeated cycling. The gradual shift of the the S-S loops along the y-axis over repeated cycles is attributed to the viscoelastic property of polymer matrix.^[1,2]^


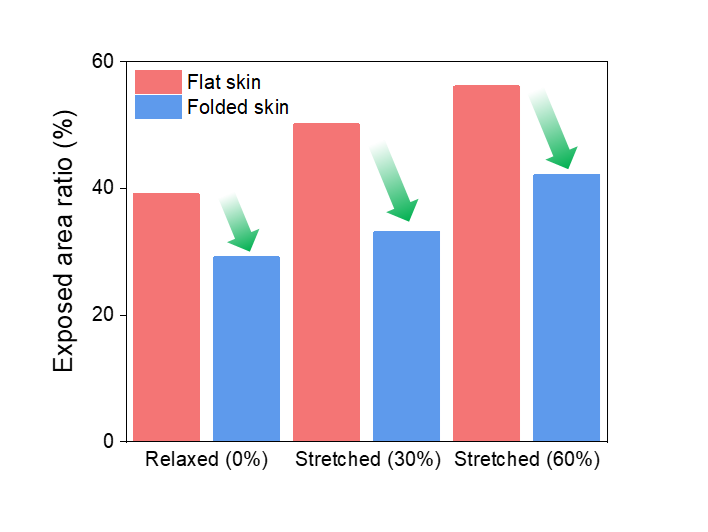


**Figure S15**. Exposed PDMS/TiO_2_ area ratio in the flat skin and folded skin with islands under stretching. Exposed area ratio (%) was calculated from top-view images as the ratio of the exposed PDMS/TiO_2_ area between the islands to the total area. The green arrows emphasize the drastic decrease in exposed area from flat to folded skin.


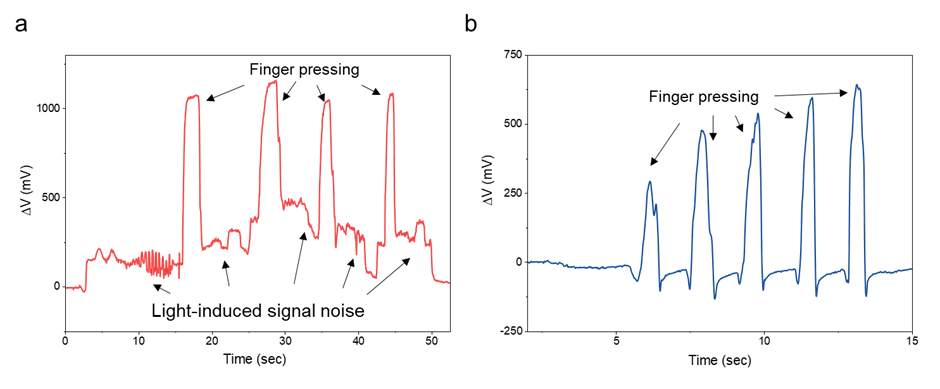


**Figure S16**. Change in voltage of optical waveguide-based sensors a) partially covered by the PDMS/TiO_2_ matrix and b) fully encapsulated with PDMS/TiO_2_ matrix pressed by finger.


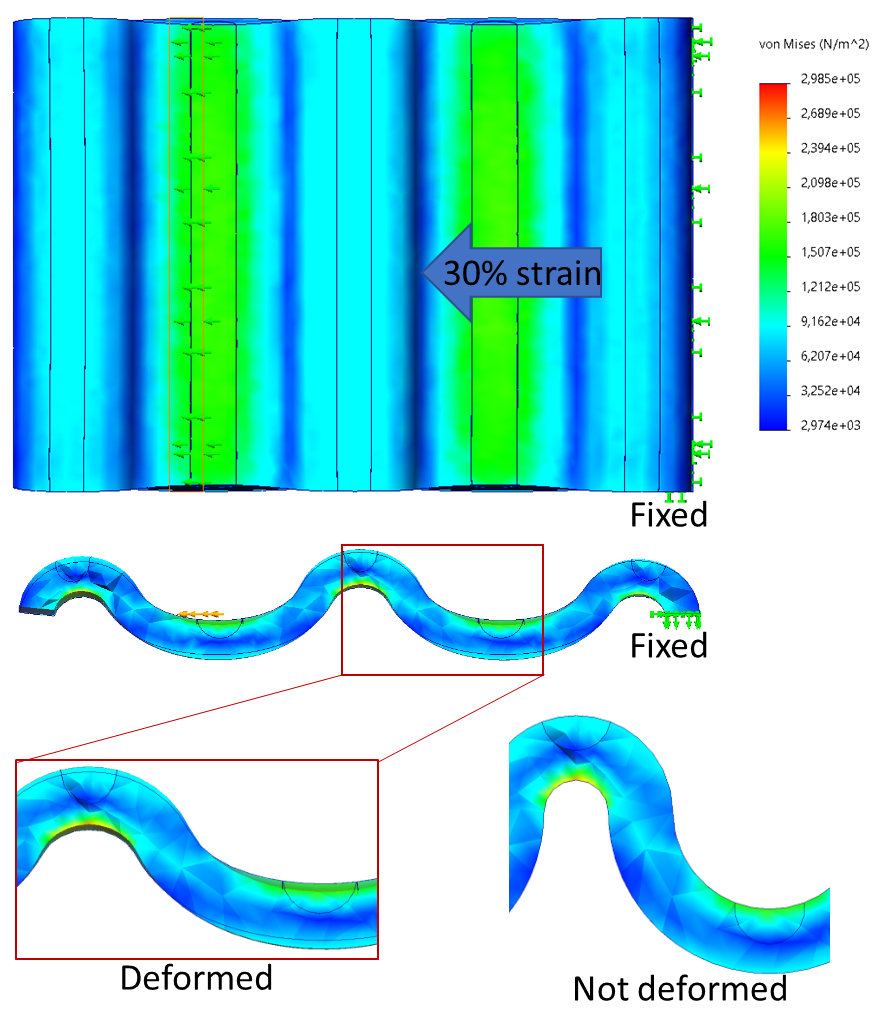


Figure S17. Finite element (FE) simulation of strain distribution in the folded skin under 30% tensile strain. The results show nonuniform strain localization along the fold structure, with higher deformation concentrated at the bottom extremum regions and reduced deformation at the top extremum regions.


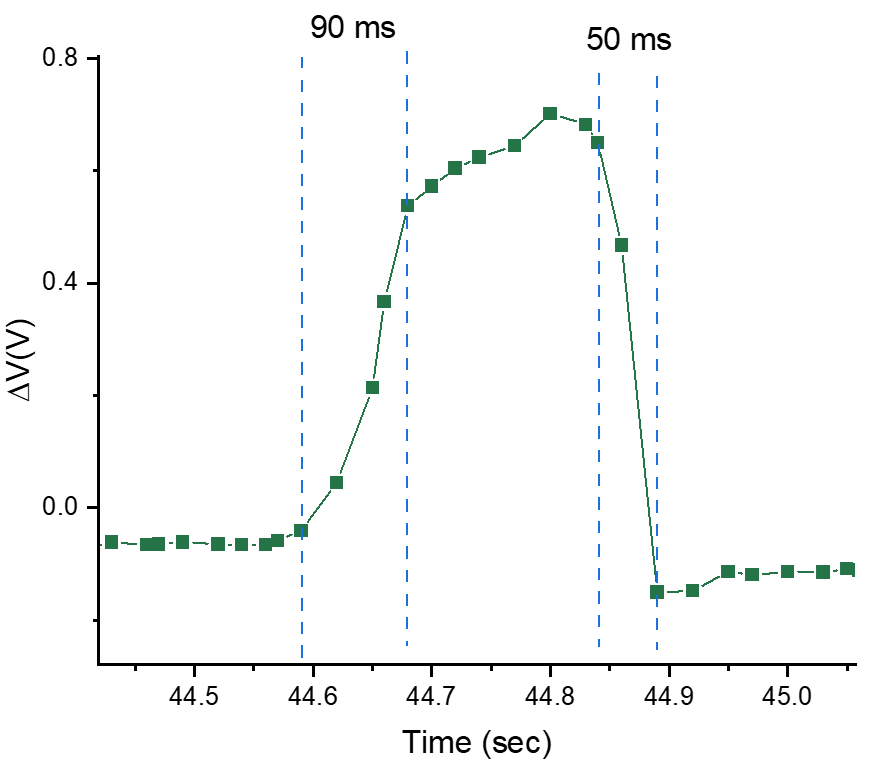


**Figure S18**. Response and recovery time of the ETATS.


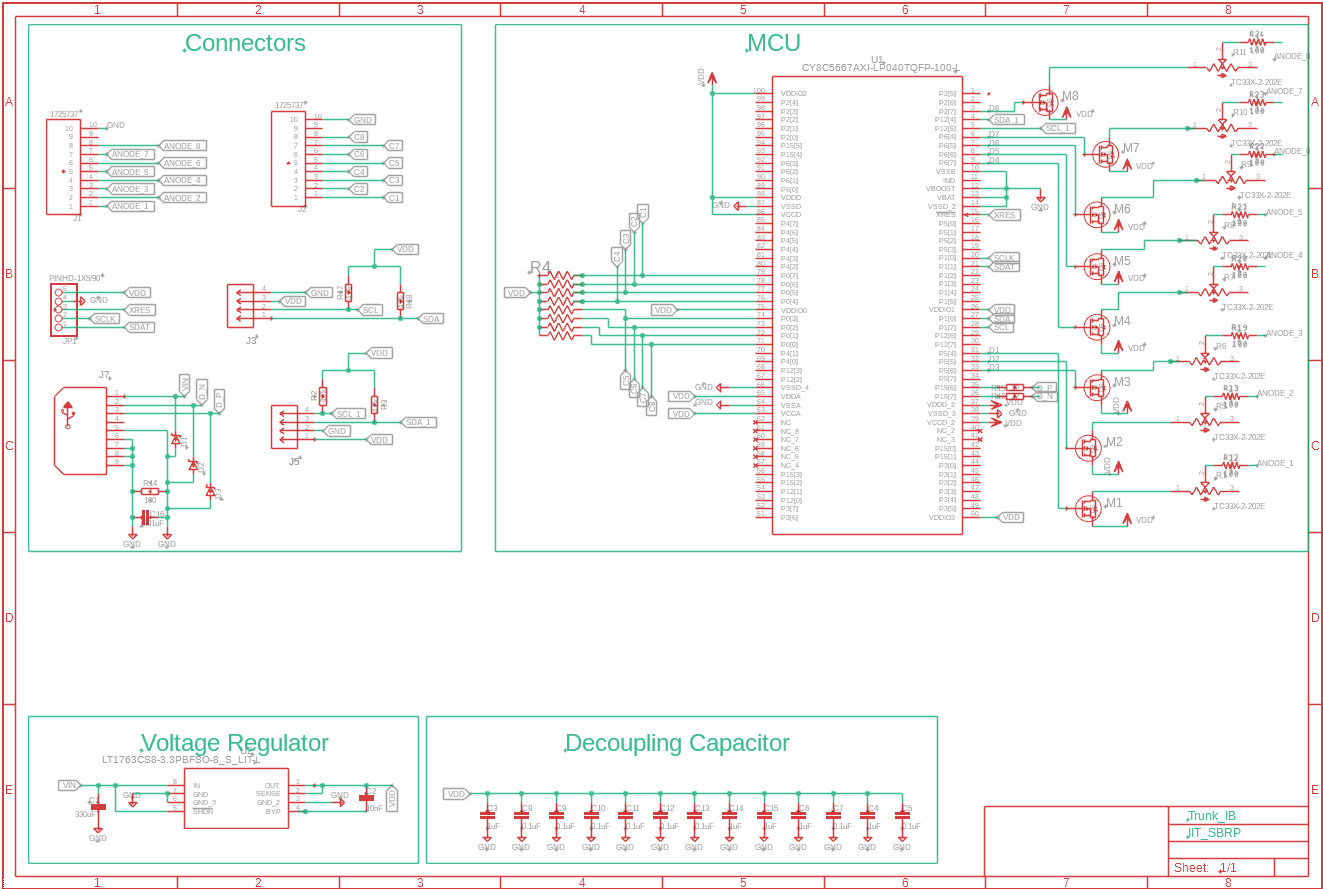


**Figure S19**. Custom PCB.

**Table S1**. A comparison of mechanical properties and tactile sensing capability of previous reported armor skins.

| **REF** | **Animal** | **Rigid**  **material** | **Base**  **materials** | **Armor**  **Performance** | **Tactile**  **sensing** | **Stretch** | **Compression** |
| --- | --- | --- | --- | --- | --- | --- | --- |
| [3] | Fish, armadillo, crocodile | Hexagon  glass | - | Puncture (~10N) | - | 0% (flexibility) | - |
| [4] | Pangolin | Hexagon  fiberglass-epoxy | PDMS | Puncture (~30N) | - | ~20% | - |
| [5] | Chiton rhyssoplax  Canariensis | 3D structured verowhiteplus®  (RGD835) | TangoBlackPlus®  (FLX980) | Puncture (>100 N) | - | 0%  (flexibility) | - |
| [6] | Snake | Modified kirigami-polyimide | - | Puncture,  scratch,  high-temperature | 0.13g ~0.53g | ~18% | - |
| [7] | Crocodile | Hexagonal epoxy islands | Fabric | Puncture,  cut resistance (5888 gf) | - | 0% (flexibility) | - |
| [8] | Fish | Overlapped alumina sheets | PU | Puncture | - | 0%  (flexibility) | - |
| **This**  **work** | **Elephant**  **trunk** | **Hexagonal epoxy**  **islands** | **Wrinkled**  **PDMS** | **Puncture**  **(97.5 N,**  **0.2mm sharp cone)**  **Puncture**  **(12.4 N,**  **25-gauge needle)**  **Cut**  **(80.5 N, knife)** | **Pressure**  **~250kPa**  **Lateral strain**  **(-30% ~+30%)** | **60%** | **40%** |

**Reference**

[1] T.-T. Yang, Y. Shui, C.-S. Wei, L.-Z. Huang, C.-W. Yang, G.-A. Sun, J.-J. Han, J.-Z. Xu, Z.-M. Li, D. Liu, *Composites Part B: Engineering* **2022,** *242*, 110100.

[2] Z. Liao, M. Hossain, X. Yao, R. Navaratne, G. Chagnon, *Polymer Testing* **2020,** *86*, 106478.

[3] R. K. Chintapalli, M. Mirkhalaf, A. K. Dastjerdi, F. Barthelat, *Bioinspir. Biomim.* **2014,** *9*, 036005.

[4] J. Choi, S. Han, M. Baliwag, B. H. Kim, H. Jang, J.-T. Kim, I. Hong, T. Kim, S. M. Kang, K.-T. Lee, D. Kang, J. A. Rogers, *Extreme Mech. Lett.* **2022,** *50*, 101537.

[5] M. Connors, T. Yang, A. Hosny, Z. Deng, F. Yazdandoost, H. Massaadi, D. Eernisse, R. Mirzaeifar, M. N. Dean, J. C. Weaver, C. Ortiz, L. Li, *Nat. Commun.* **2019,** *10*, 5413.

[6] S. Jiang, J. Liu, W. Xiong, Z. Yang, L. Yin, K. Li, Y. Huang, *Adv. Mater.* **2022,** *34*, e2204091.

[7] L. Mao, M. Zhou, L. Yao, H. Yu, X. Yan, Y. Shen, W. Chen, P. Ma, Y. Ma, S. Zhang, S. C. Tan, *Adv. Funct. Mater.* **2023,** *33*, 2213419.

[8] R. Martini, F. Barthelat, *Bioinspir. Biomim.* **2016,** *11*, 066001.
